# Hypecotumines A-D, new isoquinoline alkaloids with potential PCSK9 inhibition activity from *Hypecoum erectum* L.

**DOI:** 10.1007/s13659-024-00479-3

**Published:** 2024-10-15

**Authors:** Yinling Wei, Hongyan Wen, Lian Yang, Bodou Zhang, Xiaoyu Li, Sheng Li, Jing Dong, Zhenzhen Liang, Yu Zhang

**Affiliations:** 1 State Key Laboratory of Phytochemistry and Plant Resources in West China, Kunming Institute of Botany, Chinese Academy of Sciences, Kunming, 650201 China; 2 University of Chinese Academy of Sciences, Beijing, 100049 China

**Keywords:** *Hypecoum erectum* L., Isoquinoline alkaloids, Hypecotumines A-D, PCSK9 inhibition

## Abstract

Four new isoquinoline alkaloids, hypecotumines A-D (**1–4**), were isolated and identified from the whole herbs of *Hypecoum erectum* L. Their structures were determined by a combination of HRESIMS, NMR, and X-ray diffraction analysis methods. Compounds **1**–**4** were characterized by a terminal double bond at C-9 and their plausible biosynthetic pathway was hypothesized. Since PCSK9 plays a key role in the development of cardiovascular disease (CVD), exploration of PCSK inhibitors from natural products are beneficial for drug discovery of CVD treatment. SPR and Western blot assays showed compound **4** had PCSK9 inhibition activity with K_D_ value of 59.9 µM and thus elevated the LDLR level. Further molecular docking studies demonstrated that **4** and PCSK9 could form stable interactions via key hydrogen bonds.

**Graphical Abstract:**

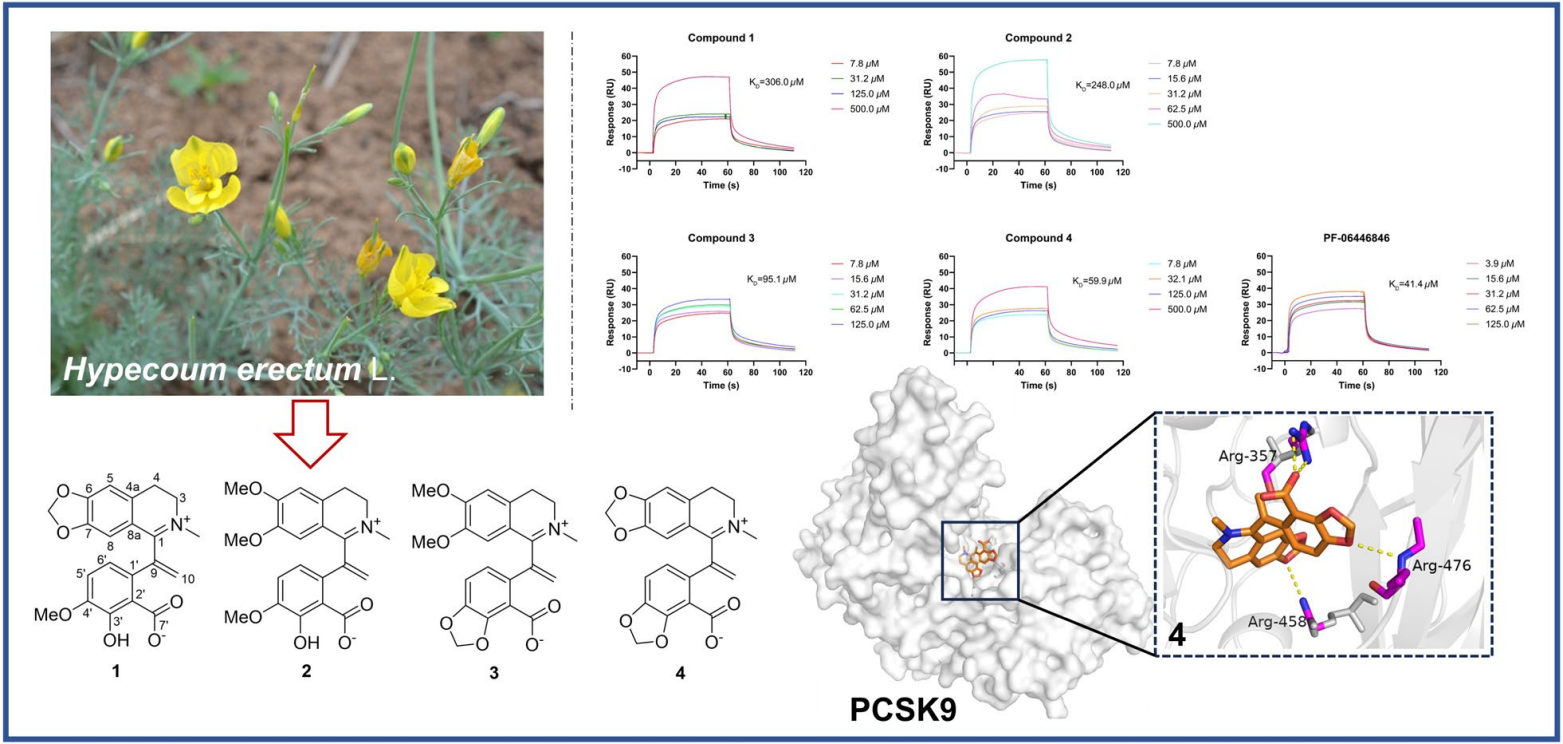

**Supplementary Information:**

The online version contains supplementary material available at 10.1007/s13659-024-00479-3.

## Introduction

Cardiovascular disease (CVD) accounts for 32% of all global deaths and remains a major threat to human health [1]. Elevated level of low-density lipoprotein cholesterol (LDL-C) is a major risk factor for atherosclerosis and cardiovascular diseases [2]. Current therapies primarily focus on reducing LDL-C levels to mitigate the risk of cardiovascular disease [2]. Statins effectively lower LDL-C levels and reduce dyslipidemia in approximately 50% of patients [3, 4]. Furthermore, a significant number of patients have exhibited intolerance and resistance to statins [5]. The discovery of functional mutations in PCSK9, which cause familial hypercholesterolemia, and the crystal structure of PCSK9 have identified it as a distinct therapeutic target for cardiovascular disease [6, 7]. PCSK9 binding to low-density lipoprotein receptor (LDL-R) disrupts the recirculation of LDL-R, thereby impairing the liver’s capacity to clear LDL-C from the bloodstream, resulting in elevated levels of LDL-C and the increased susceptibility to cardiovascular disease [8]. Hence, the intervention of PCSK9 have been identified as novel therapeutic strategies to potentially attenuate the risk of cardiovascular diseases [9].

**Fig. 1:**
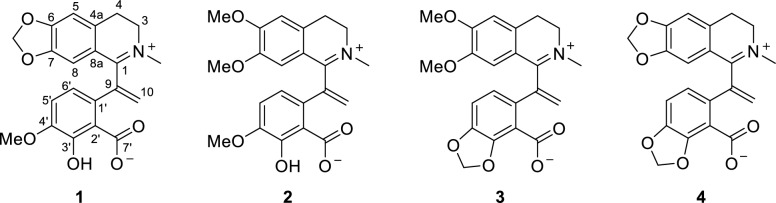
Molecular structures of compounds **1**–**4**

*Hypecoum erectum* L. is an annual herb of Papaveraceae, which is used as an ethnomedicine and local medicine. *H. erectum* is utilized as antipyretic, analgesic and anti-inflammatory substance, specifically for the treatment of fever, pharyngitis and painful conditions [10]. Current pharmaceutical studies are primarily focused on anti-inflammatory and antimicrobial aspects [11, 12], and the activity regarding cardiovascular diseases has not been reported. As our ongoing search for structural interesting and bioactive quinoline alkaloids from *H. erectum* [13], four new isoquinoline alkaloids featured terminal double bond at C-9 (Fig. 1) were isolated and identified from the whole herbs of *H. erectum*. Their affinity with PCSK9 protein were evaluated and the results suggested their potential as LDL-C lowering agents. This paper herein reported the isolation, structure elucidation, and PCSK9 inhibition activities.

## Results and discussion

Hypecotumine A (**1**) was obtained as colorless crystals in MeOH. It has the molecular formula of C_21_H_19_NO_6_, with 13 degrees of unsaturation determined by HRESIMS (*m/z* 382.1283 [M + H]^+^, calcd 382.1285) data. The IR absorption bands at 3432, 1630, and 1588 cm^−1^ indicated the presence of hydroxyl and ester carbonyl functionalities. Interpreting the ^1^H and ^13^C NMR spectra easily revealed 21 carbon atoms, which were categorized into 11 *sp*^2^ quaternary carbons, four *sp*^2^ methines, four methylenes (one *sp*^2^ methylene), and two methyl groups. These signals suggested that compound **1** was a typical isoquinoline alkaloid and had a similarity with leptopidine [14]. The distinctive differences were the presence of an additional terminal double bond (*δ*_C_ 143.4, 126.4; *δ*_H_ 5.42, 6.20) in **1**. The key HMBC correlations of H-5′ (*δ*_H_ 6.85) and H-6′ (*δ*_H_ 6.91) to *δ*_C_ 143.4 illustrated that the terminal double bond was located at C-9. The observation of HMBC cross-peaks of *δ*_H_ 6.20 and 5.42 to C-1 (*δ*_C_ 165.1) and C-1' (*δ*_C_ 130.7) further confirmed the above elucidation. 2D NMR (HSQC, HMBC, and ^1^H–^1^H COSY) experiments established the other parts of the molecule of **1** were the same as leptopidine, the planar structure of **1** was thereby established as shown in Fig. 1. 

**Fig. 2:**
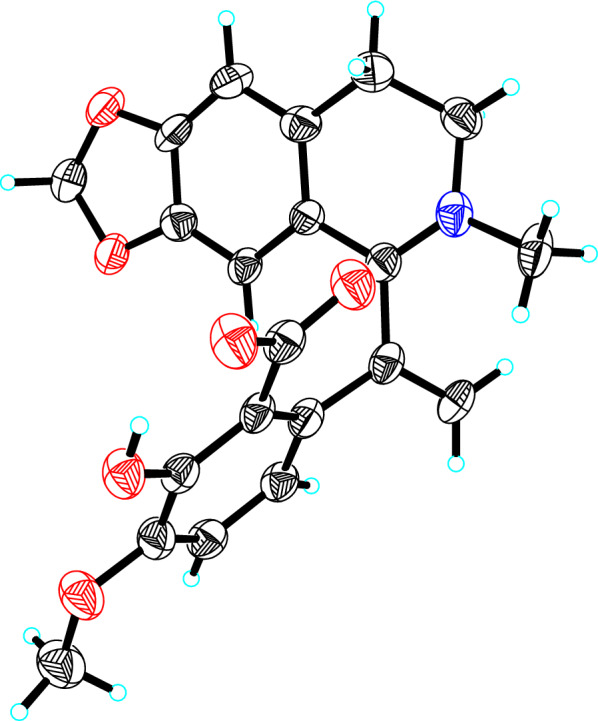
The X-ray single crystal structure of compound **1**

The existence of zwitterion in **1** was verified by X-single crystal diffraction analysis (CCDC 2348263) (Fig. 2), which unambiguously established the final structure of **1**.

**Fig. 3:**
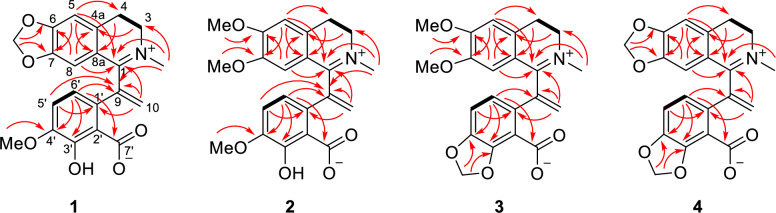
The key HMBC (arrows) and ^1^H-^1^H COSY (bold lines) correlations of compounds **1–4**

Hypecotumine B (**2**) was obtained as colorless needle crystals in MeOH and has the molecular formula of C_22_H_23_NO_6_, which was established by HRESIMS (*m/z* 398.1594 [M + H]^+^, calcd 398.1598) and ^13^C NMR data. Analyzing the ^1^H and ^13^C NMR spectroscopic data implied that compound **2** had the same structural skeleton with **1**, and the major differences were the replacement of methylenedioxy group by two methoxy groups (*δ*_C_ 55.9, 56.3; *δ*_H_ 3.56, 3.90) in **1**. The HMBC correlations of *δ*_H_ 3.56 and 3.90 to *δ*_C_ 147.4 and 154.5, respectively, suggested that the two methoxy groups were located at C-6 and C-7, respectively. Thus, the structure of **2** was assigned as shown in Fig. 1, which was verified by further 2D NMR (HSQC, HMBC, and ^1^H–^1^H COSY) experiments (Fig. 3).

Hypecotumine C (**3**) was purified as yellow oil. It had the molecular formula of C_22_H_21_NO_6_ with 13 degrees of unsaturation determined by ^13^C NMR and HRESIMS (*m/z* 396.1443 [M + H]^+^, calcd 396.1442) data. Comprehensive analysis of ^1^H and ^13^C NMR spectra suggested that compounds **3** and **2** had the same basic skeleton, and the visible distinction was the presence of a typical methylenedioxy moiety (*δ*_H_ 6.06; *δ*_C_ 102.3). The HMBC correlations of *δ*_H_ 6.06 to C-4′ (*δ*_C_ 146.1) and C-3′ (*δ*_C_ 149.3) assigned the methylenedioxy was located at C-3' and C-4'. Further analysis of HSQC, HMBC, and ^1^H–^1^H COSY spectra finally established the structure of **3** was shown in Figs. 1 and 3.

Hypecotumine D (**4**) was inferred to possess the molecular formula of C_21_H_17_NO_6_ via HRESIMS (*m/z* 380.1134 [M + H]^+^, calcd 380.1129) and ^13^C NMR data. Inspection of NMR data indicated that **4** was the analogue of compounds **1**–**3**. Two characteristic methylenedioxy moieties (*δ*_H_ 5.99, 6.09; *δ*_C_ 102.0, 101.9) situated at C-6/C-7 and C-3′/C-4′ in **4** were elucidated by HMBC correlations of *δ*_H_ 5.99 to C-6 (*δ*_C_ 151.6) and C-7 (*δ*_C_ 147.3), and of *δ*_H_ 6.09 to C-4′ (*δ*_C_ 146.7) and C-3′ (*δ*_C_ 149.3). The structure of **4** was finally confirmed by HSQC, HMBC, ^1^H–^1^H COSY spectra, as shown in Figs. 1 and 3.

**Scheme 1:**
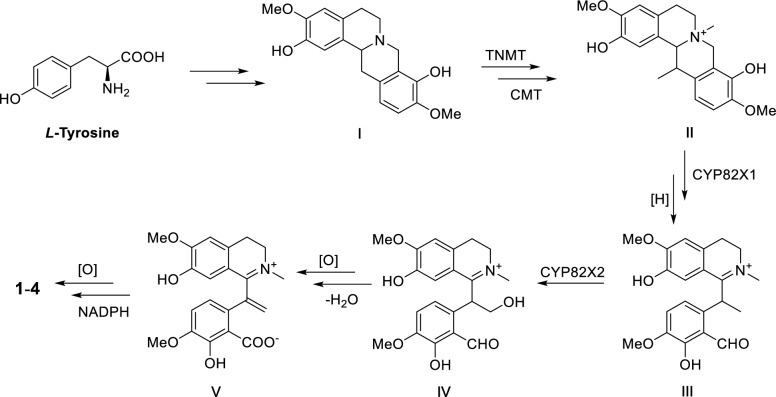
Hypothesis biogenetic pathway for compounds **1**–**4**

Since compounds **1**–**4** shared a common structural skeleton, they are likely derived from the same biosynthetic precursor (Scheme 1). *L*-tyrosine as a starting building block could form precursor I through multi-step reactions [15]. The subsequent process may be catalyzed by tetrahydroprotoberberine *N*-methyltransferase (TNMT), C-methyltransferase (CMT), and CYP82X1-like cytochrome P450 enzymes yielded key intermediate III [15–17]. Intermediate V then could be generated under the catalyzation of cytochrome p450 enzymes like CYP82X2 as the key step. Finally, the methylenedioxy moiety in compounds **1**–**4** could be formed under oxidation and NADPH as the critical steps [18].

**Fig. 4:**
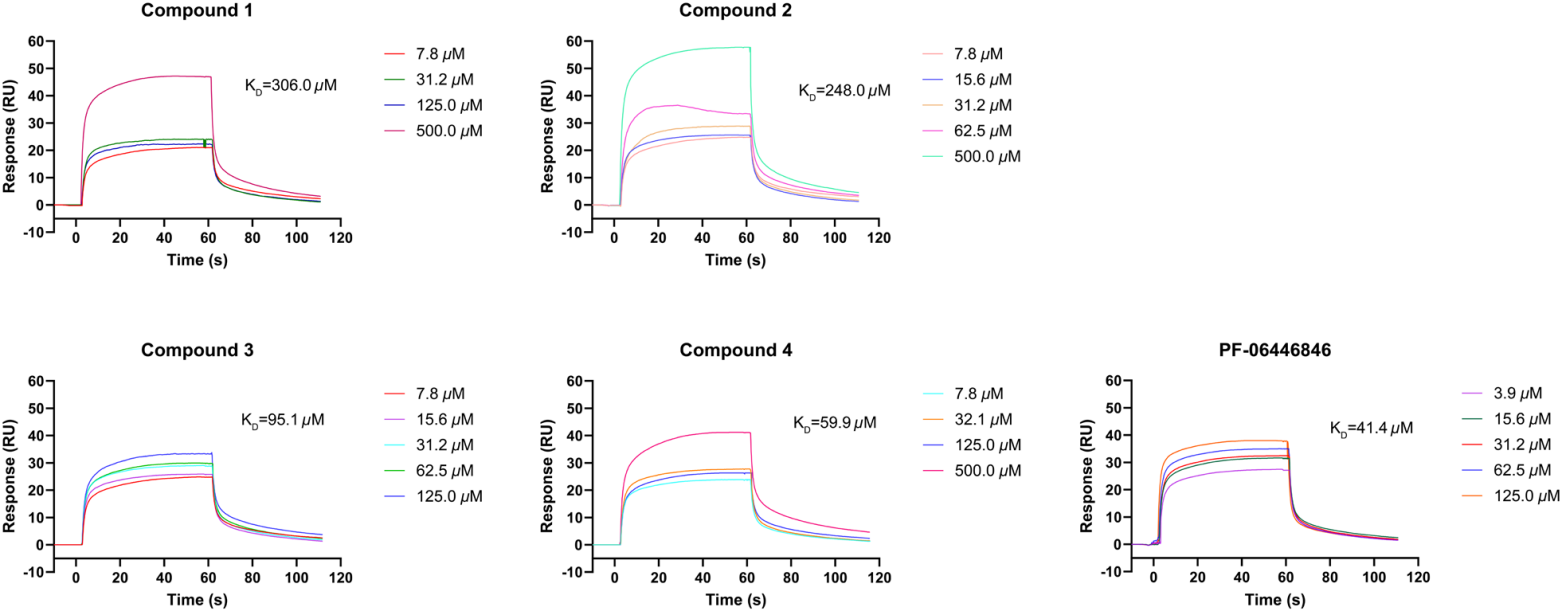
The binding affinity of compounds **1**–**4** with PCSK9 by SPR (concentrations of compounds are ranging from 7.8 to 500 µM)

**Fig. 5:**
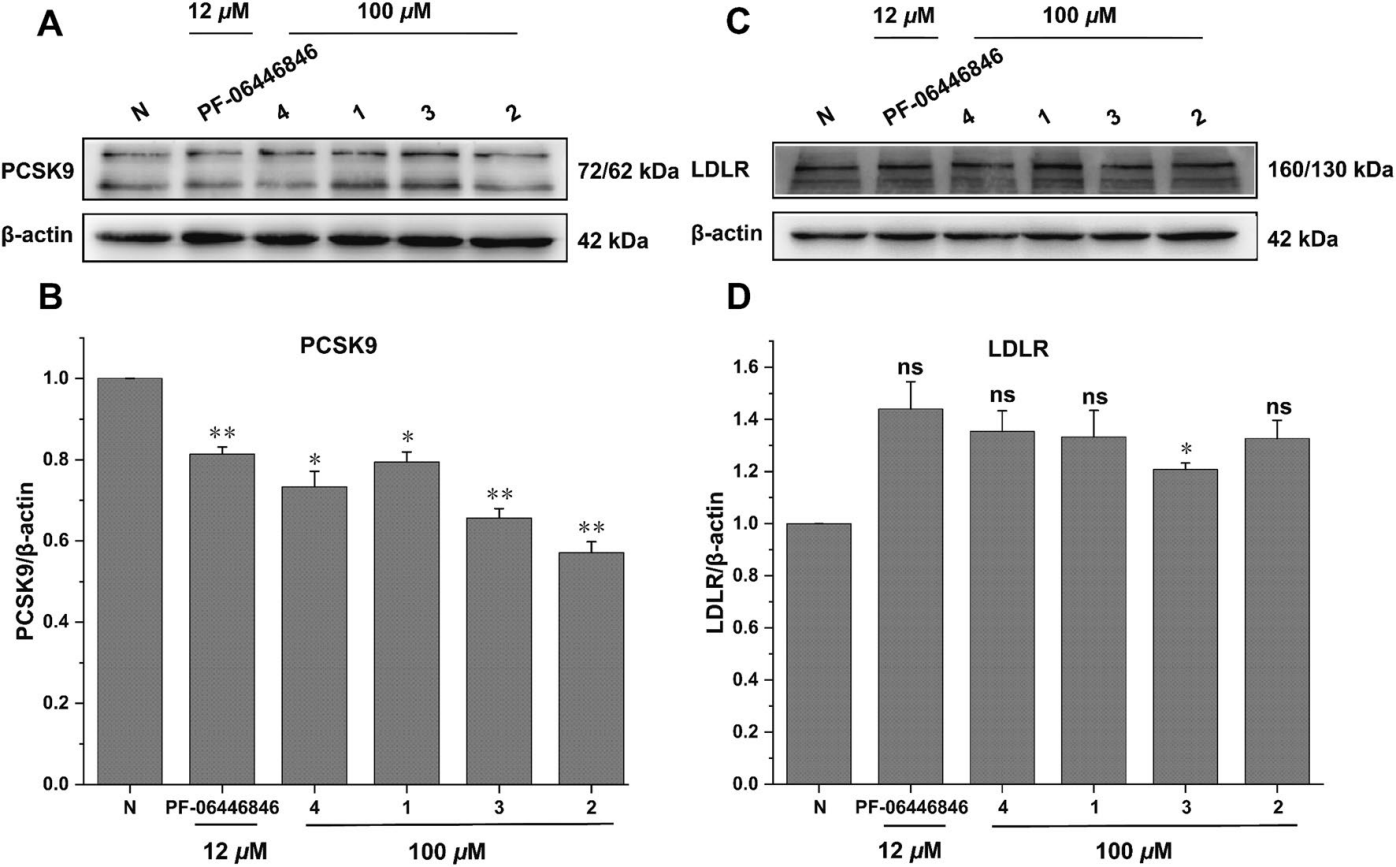
Effects of compounds **1**–**4** on PCSK9 and LDLR in HepG2 cells. (A and C) Western blotting assays showing the protein levels of PCSK9 and LDLR in HepG2 cells treated with or without compounds. (B and D) Quantification of protein levels PCSK9 and LDLR in HepG2 cells. 3 independent experiments were presented and PF-06446846 was used as a positive control. ns, not significant; *p < 0.05, **p < 0.01. One-way ANOVA with the Dunnett’s t-test. Bars represent mean ± S.E.M

All the isolates were assessed for the affinity with PCSK9 protein by surface plasmon resonance (SPR) analysis and PF-06446846 was used as the positive control [19]. The interactions were tested as the concentrations of compounds from 7.8125 to 500.0 µM with a flow rate of 10 *μ*L/min. It was found that compounds **1** and **2** had weak affinity with PCSK9, meanwhile compounds **3** and **4** had moderate affinity strength with PCSK9 compared to the positive control. The results also indicated the methylenedioxy moiety at C-3' and C-4' made a greater contribution to their affinity strength. The compound-protein interaction curves are shown in Fig. 4, and the K_D_ values of compounds **1**–**4** interacting with PCSK9 were 306.0, 248.0, 95.1, and 59.9 μM, respectively. Besides, Western blot assay was conducted to evaluate the effect of compounds **1**–**4** on protein levels of PKSC9 and LDL-R [20, 21]. The results showed that compounds **1**–**4** could downregulate the PCSK9 protein (72/62 kDa) level and thus upregulate the LDL-R protein (160/130 kDa) (Fig. 5). The results were consistent with the SPR analysis in general, which indicated compounds **1**–**4** have the potential lowering activity of LDL-C.

**Fig. 6:**
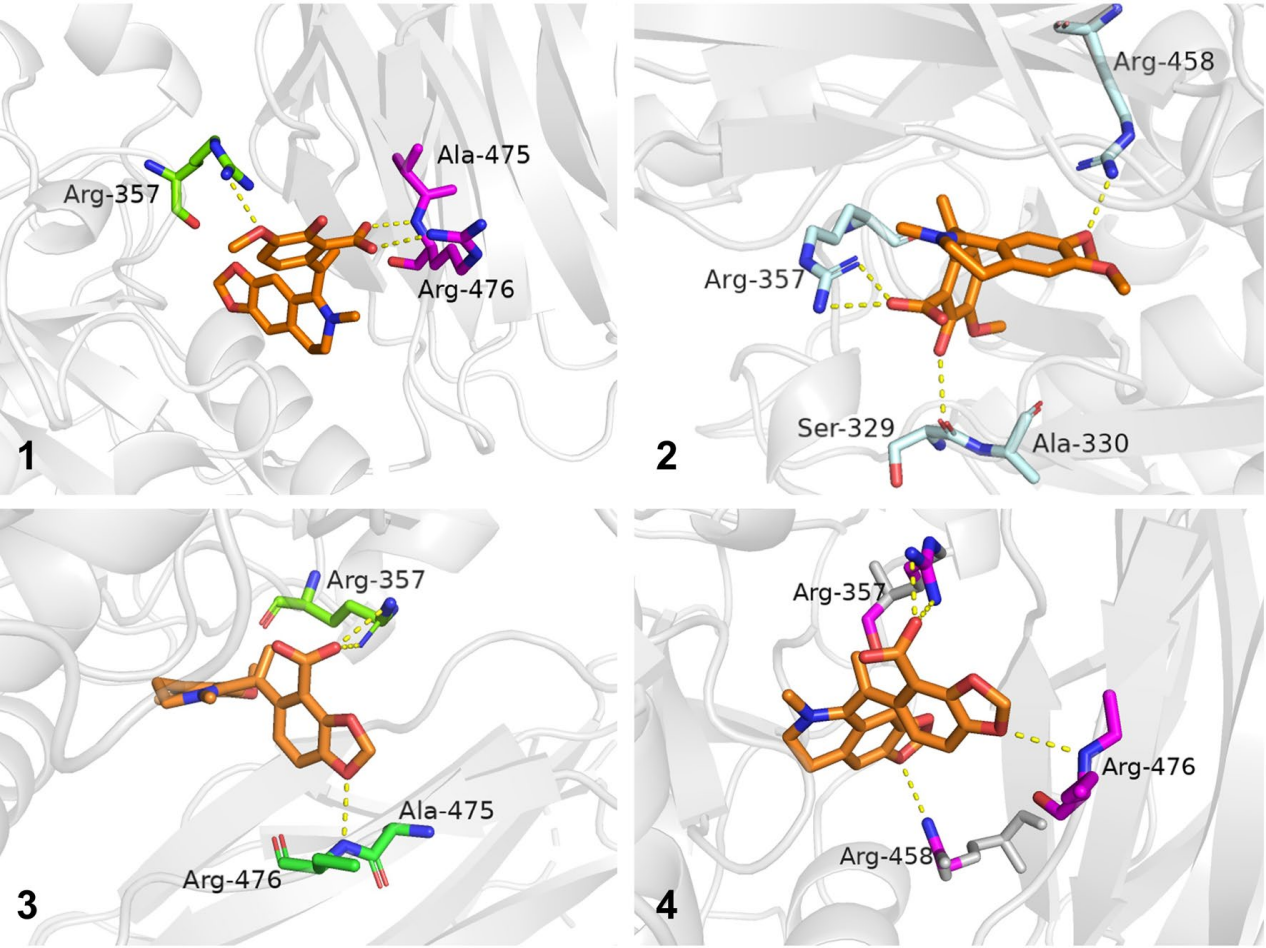
Docking results of compounds **1**–**4** with PCSK9 (PDB ID: 6U3X)

To further investigate the binding positions of compounds with the PCSK9 protein, all the compounds were docked with the crystal structure of PCSK9 protein (PDB ID: 6U3X, resolution: 2.64 Å; https://www.rcsb.org) with the software Schrodinger 2012.1, and the docking results were further processed with Pymol 3.0 [22]. The docking results revealed that all four compounds exhibited a variety of interaction forces with PCSK9, such as hydrogen bond, salt bridge and pi-cation interaction. Some of the key interactions between compounds and amino acid residues (e.g. ARG-357 and ARG-458) were same as those of C4 [22]. Comparing to docking result of PF-06446846, there were also pi-cation interaction with ARG-458 and salt bridge with ASP-360 in compound **4**. However, these interactions were absent in compounds **1**–**3**, which might be the reason that **4** had better affinity with PCSK9 (Figure S5). Docking results are shown in Fig. 6, the yellow dotted lines represent interaction forces.

## Experimental

### General experimental procedures

SPR analysis were performed on Biacore T200. X-ray data were recorded using a Bruker APEX DUO instrument. NMR, HRESIMS, UV, and IR spectra were measured as described previously [13].

### Plant material

*H. erectum* (whole herbs) was bought from Eastern medicinal materials market of Hebei Province in September 2023. The voucher specimen (No. KIBZY20230702) was identified by Dr. Chunlei Xiang and preserved at Kunming Institute of Botany.

### Extraction and isolation

Crude alkaloids (800 g) were obtained from *H. erectum* whole herbs (40.0 kg) by following the published procedures [13], which were separated into five fractions (I-V) by silica gel CC eluted with CH_2_Cl_2_/MeOH (60:1 → 0:1, v/v). Fr. V (30.0 g) was eluted with CH_2_Cl_2_/MeOH (40:1 → 0:1, v/v) to give five subfractions (V1-5). Fr. V5 (6.0 g) was subjected to Sephadex LH-20 (MeOH), followed by semipreparative high-performance using Waters X-Bridge C18 (10 × 150 mm, 5 μm) column with CH_3_CN/H_2_O (32:68, 0.1%, v/v, formic acid) to afford compound** 3** (8.5 mg, t_*R*_ = 23.0 min). Fr. V4 (12.3 g) was subjected to gradient elution (CH_2_Cl_2_/MeOH: 30:1 → 0:1, v/v) and followed by semi-preparative high performance liquid chromatography (HPLC) separation using Waters X-Bridge C18 (10 × 150 mm, 5 μm) column, CH_3_CN/H_2_O (38:62, 0.1%, v/v, formic acid) to afford compound **4** (5.1 mg, t_R_ = 42.0 min). Fr. IV (80.0 g) was eluted with CH_2_Cl_2_/MeOH (50:1 → 0:1, v/v) to give five subfractions (IV1-5). IV3 (15.3 g) was then subjected to gradient elution (CH_2_Cl_2_/MeOH: 30:1 → 0:1, v/v), purified using semi-preparative HPLC with Waters X-Bridge C18 (10 × 150 mm, 5 μm) column CH_3_CN/H_2_O (48:52, 0.1%, v/v, formic acid), which finally gave compounds **1** (6.2 mg, t_R_ = 32.6 min), and **2** (26.8 mg, t_R_ = 17.5 min).

### Compound characterization

*Hypecotumine A (****1****)*: white crystal; UV (MeOH) *λ*_max_ (log *ε*) 210.0 (5.38), 241.5 (5.12), 312.0 (4.78), 357.5 (4.75) nm; IR (KBr) *ν*_max_ 3432, 2912, 1630, 1588, 1504, 1485,1394 and 1366 cm^−1^; ESIMS *m/z* 382 [M + H]^+^; HRESIMS *m/z* 382.1283 [M + H]^+^ (calcd for C_21_H_19_NO_6_, 382.1285); ^1^H and ^13^C NMR data, see Tables 1 and 2, respectively.

**Table 1:** ^1^H NMR (500 MHz) spectroscopic data for compounds **1**–**4** (*δ* in ppm, *J* in Hz)

Position	**1**^a^	**2**^a^	**3**^a^	**4**^a^
3	3.90 (2H, m)	3.15 (2H, m)	3.48 (2H, m)^b^	3.42 (2H, m)
4	3.02 (2H, m)	2.94 (2H, m)	3.12 (2H, m)^b^	2.73 (2H, m)
5	6.64 (1H, s)	6.66 (1H, s)	6.70 (1H, s)	6.68 (1H, s)
8	7.06 (1H, s)	7.04 (1H, s)	6.91 (1H, s)	6.85 (1H, s)^b^
10a	5.42 (1H, s)	5.47 (1H, s)	5.50 (1H, s)	5.50 (1H, s)
10b	6.20 (1H, s)	6.22 (1H, s)	6.28 (1H, s)	6.26 (1H, s)
5'	6.91 (1H, d, 8.0)	6.88 (1H, d, 8.0)	6.84 (1H, d, 8.0)	6.86 (1H, d, 8.0)^b^
6'	6.85 (1H, d, 8.0)	6.85 (1H, d, 8.0)	7.06 (1H, d, 8.0)	7.10 (1H, d, 8.0)
OCH_2_O	5.98 (2H, s)			5.99 (2H, s)
OCH_2_O'			6.06 (2H, s)	6.09 (2H, s)
6-OMe		3.90 (3H, s)	3.91 (3H, s)	
7-OMe		3.56 (3H, s)	3.63 (3H, s)	
4'-OMe	3.90 (3H, s)	3.88 (3H, s)		
*N*Me	3.35 (3H, s)	3.62 (3H, s)	3.47 (3H, s)	3.49 (3H, s)

^a^Measured in CDCl_3_

^b^Overlapped

**Table 2:** ^13^C NMR (125 MHz) spectroscopic data for compounds **1**–**4** (*δ* in ppm)

Position	**1**	**2**	**3**	**4**
1	165.1	170.5	166.7	165.6
3	51.9	52.5	51.3	52.5
4	26.8	26.0	26.1	27.1
4a	134.3	133.2	132.5	130.7
5	107.2	109.6	110.2	110.0
6	146.7	154.5	154.3	151.6
7	152.5	147.4	147.9	147.3
8	112.7	115.3	114.0	110.2
8a	123.7	120.6	121.7	121.8
9	143.4	143.8	140.7	140.5
10	126.4	126.3	124.0	128.2
1'	130.7	131.7	129.3	128.9
2'	115.1	116.2	119.2	120.2
3'	154.0	154.3	149.3	149.3
4'	150.2	150.3	146.1	146.7
5'	113.2	112.2	109.0	107.9
6'	118.1	118.8	121.0	120.0
7'	172.1	172.4	174.2	173.2
OCH_2_O	102.2			102.0
OCH_2_O′			102.3	101.9
6-OMe		56.3	56.3	
7-OMe		55.9	56.0	
4'-OMe	55.9	55.9		
*N*Me	45.0	46.0	44.3	44.8

Measured in CDCl_3_

*Hypecotumine B (****2****)*: white needle-like crystal, amorphous powder; UV (MeOH) *λ*_max_ (log *ε*) 208.5 (5.23), 240.0 (4.95), 314.0 (4.69), 355.0 (4.59) nm; IR (KBr) *ν*_max_ 3432, 2937, 1626, 1606, 1564, 1519, 1481 and 1461 cm^−1^; ESIMS *m/z* 398 [M + H]^+^; HRESIMS m/z 398.1594 [M + H]^+^ (calcd for C_22_H_23_NO_6_, 398.1598); ^1^H and ^13^C NMR data, see Tables 1 and 2, respectively.

*Hypecotumine C (****3****)*: yellow oil; UV (MeOH) *λ*_max_ (log *ε*) 204.5 (5.06), 242.5 (4.69), 304.5 (4.43), 372.0 (4.30) nm; IR (KBr) *ν*_max_ 3432, 2932, 1709, 1604, 1564, 1519, 1444 and 1382 cm^−1^; ESIMS m/z 396 [M + H]^+^; HRESIMS m/z 396.1443 [M + H]^+^ (calcd for C_22_H_21_NO_6_, 396.1442); ^1^H and ^13^C NMR data, see Tables 1 and 2, respectively.

*Hypecotumine D (****4****)*: yellow oil; UV (MeOH) *λ*_max_ (log *ε*) 206.0 (5.49), 245.5 (5.12), 300.5 (4.84), 373.0 (4.66) nm; IR (KBr) *ν*_max_ 3434, 2924, 1630, 1607, 1503, 1486 1465 and 1384 cm^−1^; ESIMS *m/z* 380 [M + H]^+^; HRESIMS *m/z* 380.1134 [M + H]^+^ (calcd for C_21_H_17_NO_6_, 380.1129); ^1^H and ^13^C NMR data, see Tables 1 and 2, respectively.

### Crystal data for *Hypecotumine A (1)*

*Hypecotumine A (****1****)*: C_21_H_19_NO_6_•3(H_2_O), *M* = 435.42, *a* = 40.812(3) Å, *b* = 13.2173(9) Å, *c* = 15.3426(9) Å, *α* = 90°, *β* = 90.934(7)°, *γ* = 90°, *V* = 8275.1(9) Å^3^, *T* = 150.(2) K, space group *C*12*/c*1, *Z* = 16, *μ*(Cu Kα) = 0.931 mm^−1^, 36,414 reflections measured, 7593 independent reflections (*R*_*int*_ = 0.3067). The final *R*_*1*_ values were 0.1195 (*I* > 2*σ*(*I*)). The final *wR*(*F*^2^) values were 0.3090 (*I* > 2*σ*(*I*)). The final *R*_*1*_ values were 0.1990 (all data). The final *wR*(*F*^2^) values were 0.3769 (all data). The goodness of fit on *F*^2^ was 0.996. Crystallographic data (excluding structure factor tables) for compound **1** have been deposited with the Cambridge Crystallographic Data Center as supplementary publication (deposit number CCDC 2348263). Copies of the data can be obtained free of charge by application to CCDC, 12 Union Road, Cambridge CB 1EZ, UK [fax: Int. + 44 (0) (1223) 336033; e-mail: deposit@ccdc.cam.ac.uk.

### Experimental methods

Protein preparation: The protein concentration of PCSK9 was 40 µg/mL. Ligand pre-enrichment: ligand pre-enrichment was performed by manual injection to test the pre-enrichment of PCSK9 at a concentration of 40 µg/mL in 10 mM sodium acetate solution at pH 4.5 and 5.0 (pH 4.5 was selected for coupling). The proteins were immobilized in the Fc-4 channel of CM5 chip using amino coupling, respectively. And the Fc-1 channel was activated and closed as the reference channel of the Fc-4 channel, respectively [23].

### Compounds interaction analysis with protein PCSK9

Biacore T200 was used to characterize interactions between small molecules and protein. The compounds **1–4** were formulated in a concentration gradient using running buffer to prepare a series of concentrations ranging from 500.0, 250.0, 125.0, 62.5, 31.25, 15.625, and 7.8125 µM. Flow through Fc1-Fc4 and determine the interaction between compounds and PCSK9 immobilized on the chip surface. Operation parameter setting: flow path 4–1 (contact time 80 s, flow rate 30 *μ*L/min, dissociation time 80 s), 25 ℃.

### Cell culture

Human hepatocellular liver cell, HepG2 was obtained from ATCC Cell Bank and cultured 12–24 h in advance in RMPI1640 medium using culture medium containing 10% fetal bovine serum. Positive drug and compounds were solubilized using DMSO and diluted to 12 µmol and 100 µmol individually. Then the compounds were added to the cells 6 well plates, incubated in a 37 °C cell culture incubator at 5% CO_2_ for 24 h.

### Western blot assay

HepG2 cells were washed two times in pre-cooled PBS and lysed using RIPA buffer (Thermo fisher CN) on ice. The cells were centrifuged for 10 ~ 15 s per 10 min to fully lyse, continuing 60 min, and detected the protein concentration with BCA protein concentration assay kit. Proteins were separated by 12% sodium dodecyl sulfate polyacrylamide gel (SDS-PAGE) with electrophoresis and transferred to activated PVDF membrane (Millipore USA). Then, PVDF membrane were treated with 5% milk for 1.5 h. After that, the membrane was incubated with primary antibody (1:500) overnight at 4 °C and with secondary antibody (1:5000) for 60 min at room temperature. The band pictures were got by Gel imaging and chemiluminescence imaging system (UVITEC UK).

### Statistical analysis

The experiment was repeated five times and the results were expressed by mean ± S.E.M. Significance level was fixed by one-way analysis of variance (ANOVA) and Dunnett’s t-test. P represented the degree of significance [20].

### Molecular docking

Schrodinger 2012.1 soft was used for molecular docking. The docking results were processed by Pymol. The PDB of PCSK9 protein is 6U3X (resolution: 2.64 Å) (https://www.rcsb.org). The process of protein preparation includes addition of hydrogens, addition of missing side chains, removal of waters, assigning protonation states and energy minimization [24]. Ligands were prepared by generating 3D coordinates, using OPLS3e force field, assigning protonation states, and energy minimization formation. The position and size of the docking box was determined by the position and size of the original ligand. The SP docking mode was used to generate docking poses.

## Concluding remarks

Four new isoquinoline alkaloids, hypecotumines A-D (**1–4**), featured terminal double bond at C-9 were isolated and identified from *H. erectum*. These alkaloids were evaluated for their affinity with PCSK9 by SPR assay and were further verified by Western blot assay, the results showed alkaloids **1**–**4** had potential PCSK9 inhibition activity. Further molecular docking verified the intimate interaction of alkaloids **1–4** and PCSK9, which demonstrated that alkaloids **1–4** are expected to be lead compound for LDL-C lowering.

## Supplementary Information

Supplementary material 1.

## Data Availability

The data supporting the results of this study can be obtained from the corresponding author upon reasonable request.
