# Supplementary material for: Hypecotumines A-D, new isoquinoline alkaloids with potential PCSK9 inhibition activity from Hypecoum erectum L
Source: Nat Prod Bioprospect. 2024 Oct 15;14(1):57. doi: 10.1007/s13659-024-00479-3 (PMC11480295; doi:10.1007/s13659-024-00479-3)
Supplement: Supplementary file 1 — Supplementary material 1. [file 13659_2024_479_MOESM1_ESM.pdf]

SUPPORTING INFORMATION

Hypecotumines A-D, new isoquinoline alkaloids with potential PCSK9 inhibition activity from *Hypecoum erectum* L.

Yinling Wei [a,b], Hongyan Wen [a], Lian Yang [a] Bodou Zhang [a,b], Xiaoyu Li [a,b], Sheng Li [a], Jing Dong [a], Zhenzhen Liang [a], Yu Zhang [a,*]

[a] *State Key Laboratory of Phytochemistry and Plant Resources in West China, Kunming Institute of Botany, Chinese Academy of Sciences, Kunming 650201, China*

[b] *University of Chinese Academy of Sciences, Beijing, 100049, PR China*

Content

Figure S1.1 ^{1}H NMR (500 MHz, $CDCl_3$) of **1**.

Figure S1.2 ^{13}C NMR (125 MHz, $CDCl_3$) of **1**.

Figure S1.3 HSQC (500 MHz, $CDCl_3$) of **1**.

Figure S1.4 HMBC (500 MHz, $CDCl_3$) of **1**.

Figure S1.5 ^{1}H-^{1}H COSY (500 MHz, $CDCl_3$) of **1**.

Figure S1.6 HRESIMS spectrum of **1**.

Figure S1.7 IR (KBr disk) spectrum of **1**.

Figure S1.8 X-ray crystal structure of **1**.

Figure S2.1 ^{1}H NMR (500 MHz, $CDCl_3$) of **2**.

Figure S2.2 ^{13}C NMR (125 MHz, $CDCl_3$) of **2**.

Figure S2.3 HSQC (500 MHz, $CDCl_3$) of **2**.

Figure S2.4 HMBC (500 MHz, $CDCl_3$) of **2**.

Figure S2.5 ^{1}H-^{1}H COSY (500 MHz, $CDCl_3$) of **2**.

Figure S2.6 HRESIMS spectrum of **2**.

Figure S2.7 IR (KBr disk) spectrum of **2**.

Figure S3.1 ^{1}H NMR (500 MHz, $CDCl_3$) of **3**.

Figure S3.2 ^{13}C NMR (125 MHz, $CDCl_3$) of **3**.

Figure S3.3 HSQC (500 MHz, $CDCl_3$) of **3**.

Figure S3.4 HMBC (500 MHz, $CDCl_3$) of **3**.

Figure S3.5 ^{1}H-^{1}H COSY (500 MHz, $CDCl_3$) of **3**.

Figure S3.6 HRESIMS spectrum of **3**.

Figure S3.7 IR (KBr disk) spectrum of **3**.

Figure S4.1 ^{1}H NMR (500 MHz, $CDCl_3$) of **4**.

Figure S4.2 ^{13}C NMR (125 MHz, $CDCl_3$) of **4**.

Figure S4.3 HSQC (500 MHz, $CDCl_3$) of **4**.

Figure S4.4 HMBC (500 MHz, $CDCl_3$) of **4**.

Figure S4.5 ^{1}H-^{1}H COSY (500 MHz, $CDCl_3$) of **4**.

Figure S4.6 HRESIMS spectrum of **4**.

Figure S4.7 IR (KBr disk) spectrum of **4**.

Figure S5. The 2D interacting mode of docking results of compounds **1**-**4** and PF-06446846 with PCSK9 (PDB ID: 6U3X).

Figure S1.1 ^{1}H NMR (500 MHz, $CDCl_3$) of **1**

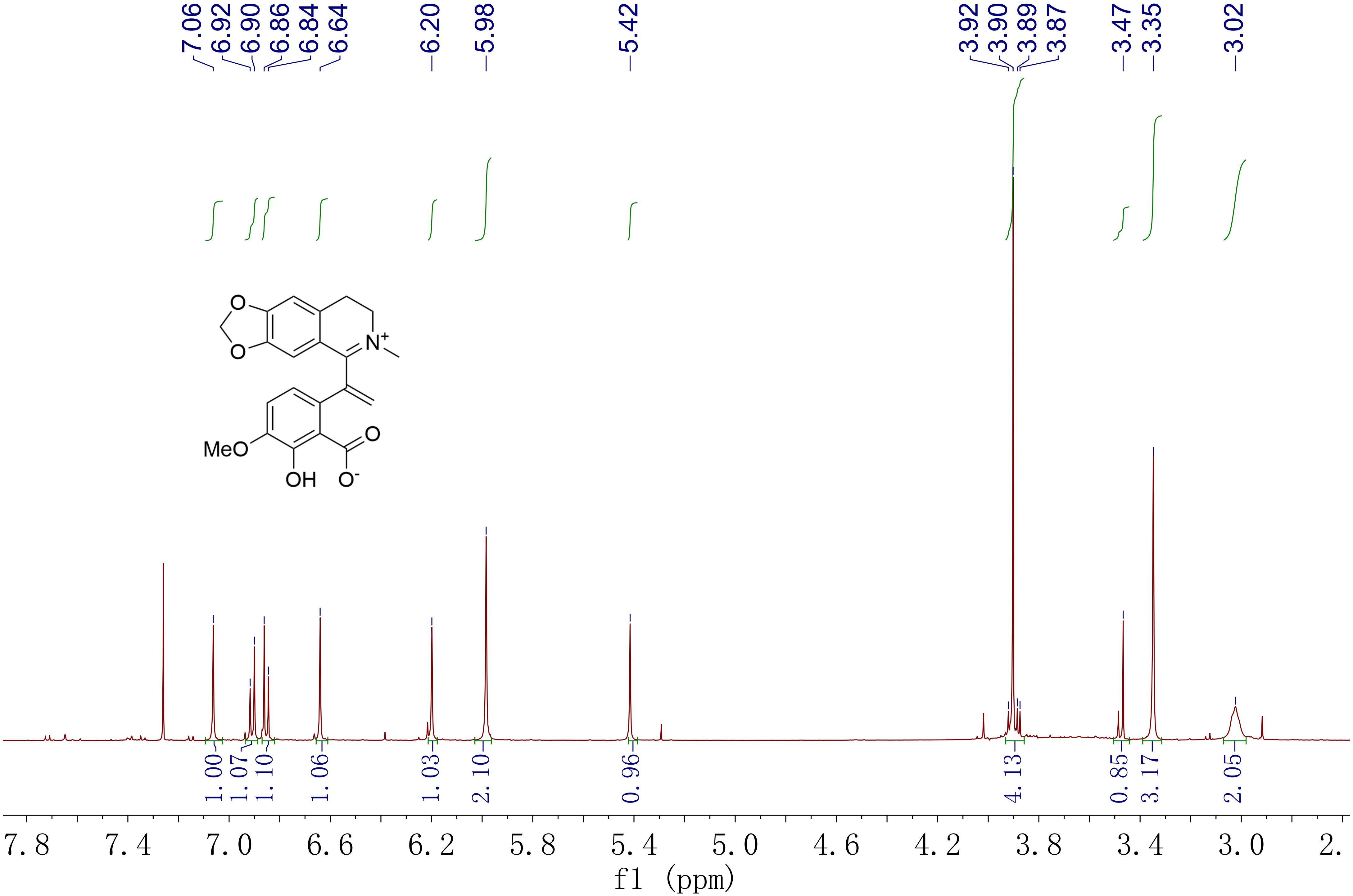

Figure S1.2 ^{13}C NMR (125 MHz, $CDCl_3$) of **1**

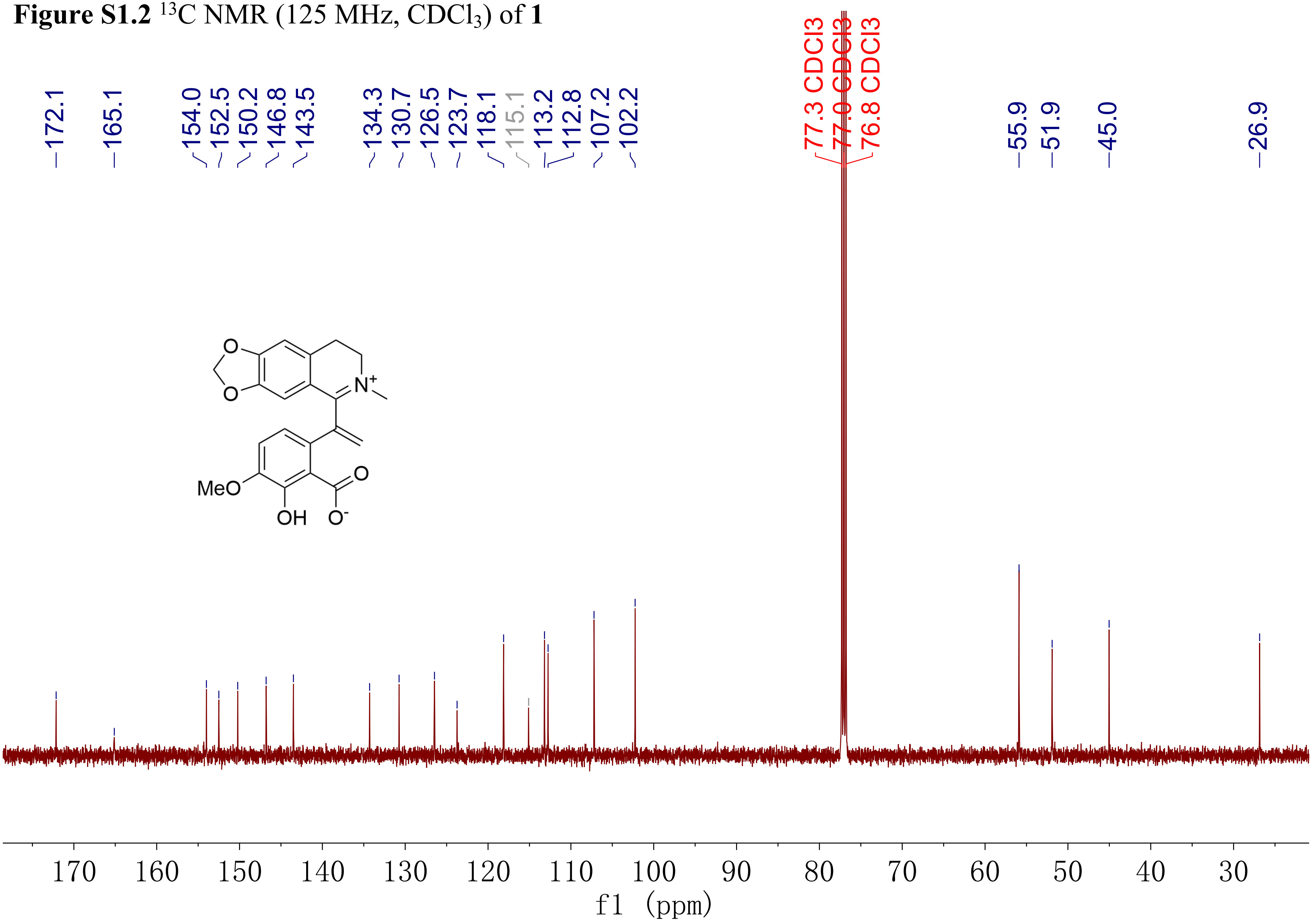

Figure S1.3 HSQC (500 MHz, $CDCl_3$) of **1**

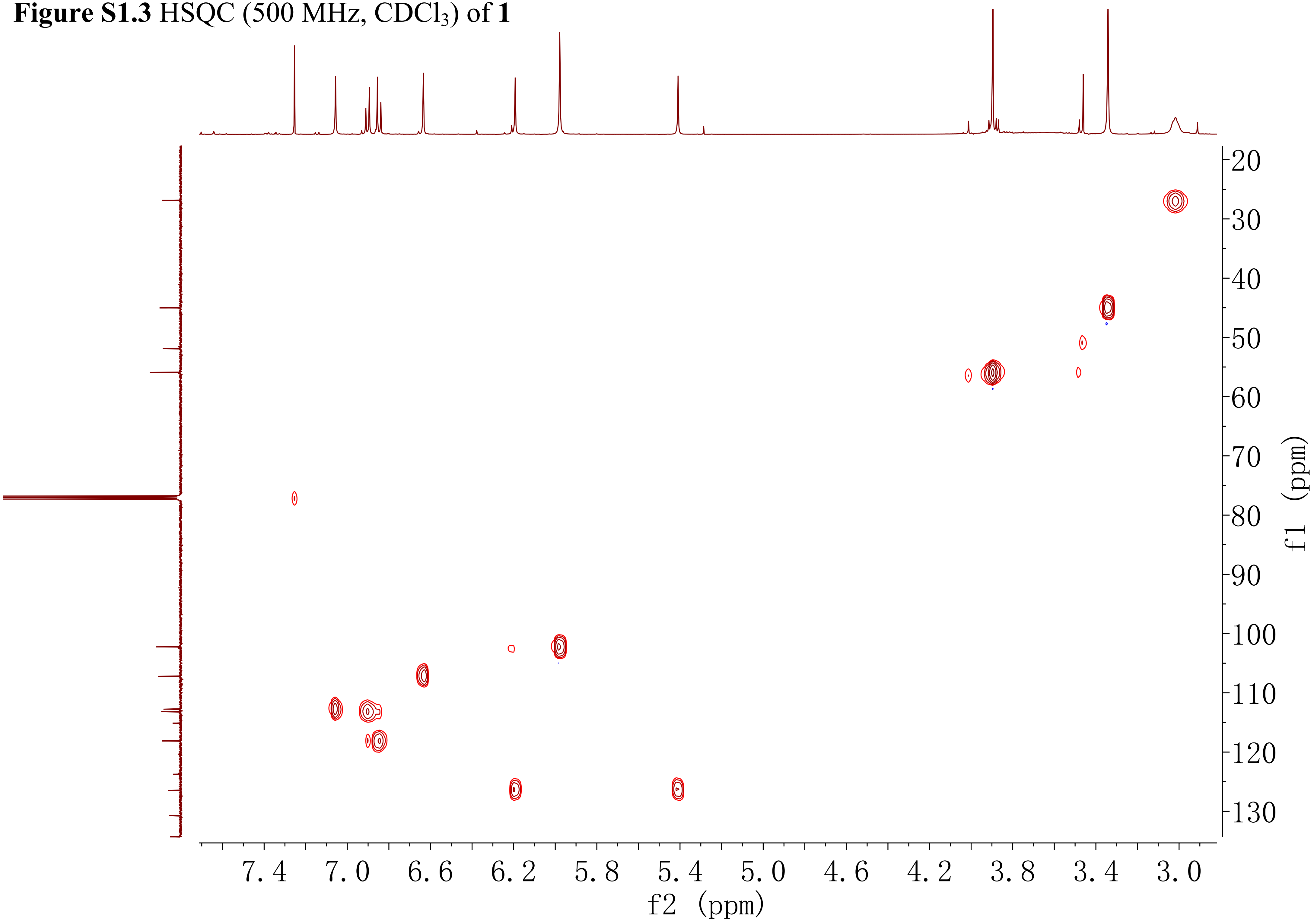

Figure S1.4 HMBC (500 MHz, $CDCl_3$) of **1**

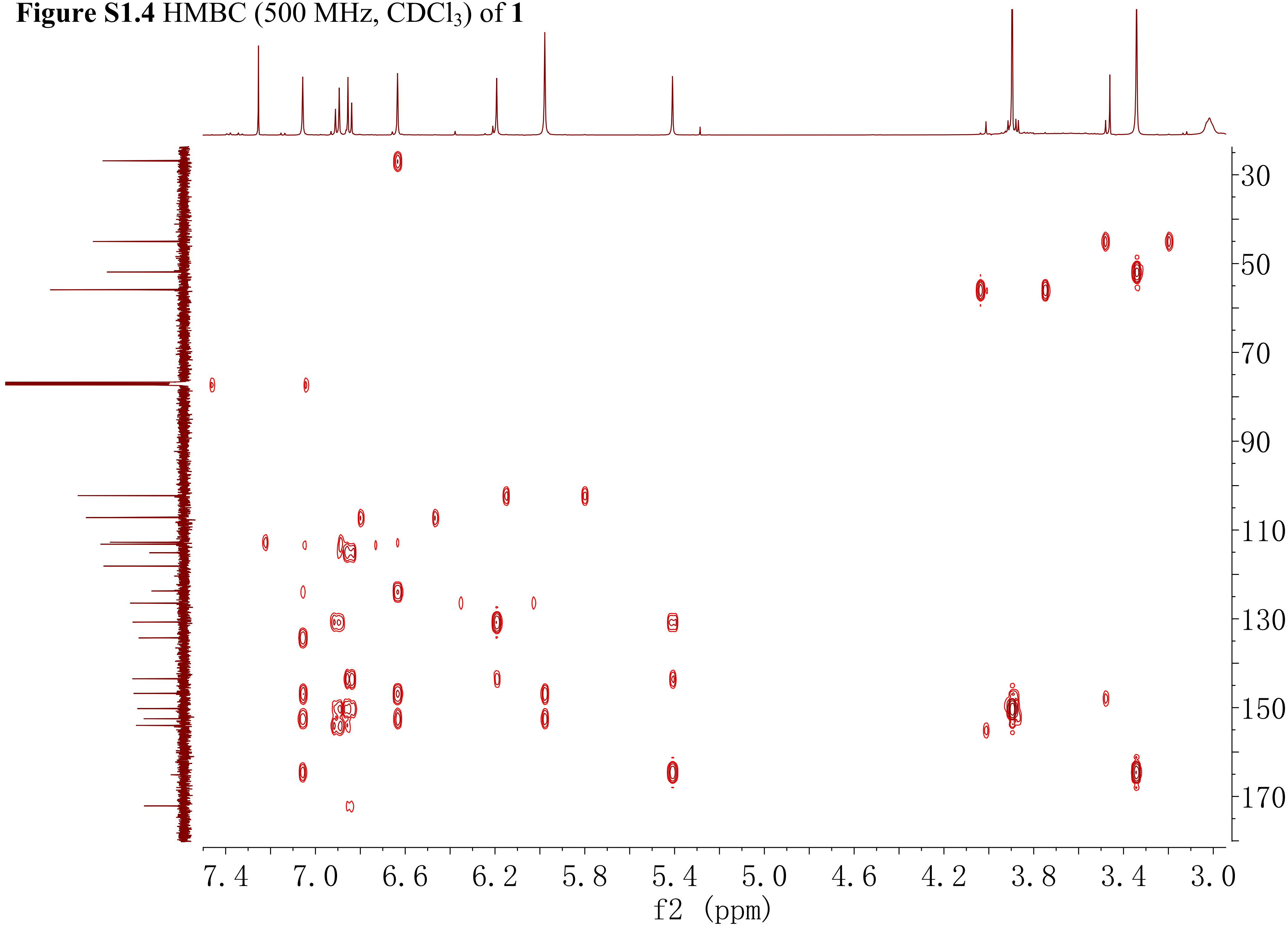

Figure S1.5 ^{1}H-^{1}H COSY (500 MHz, $CDCl_3$) of **1**

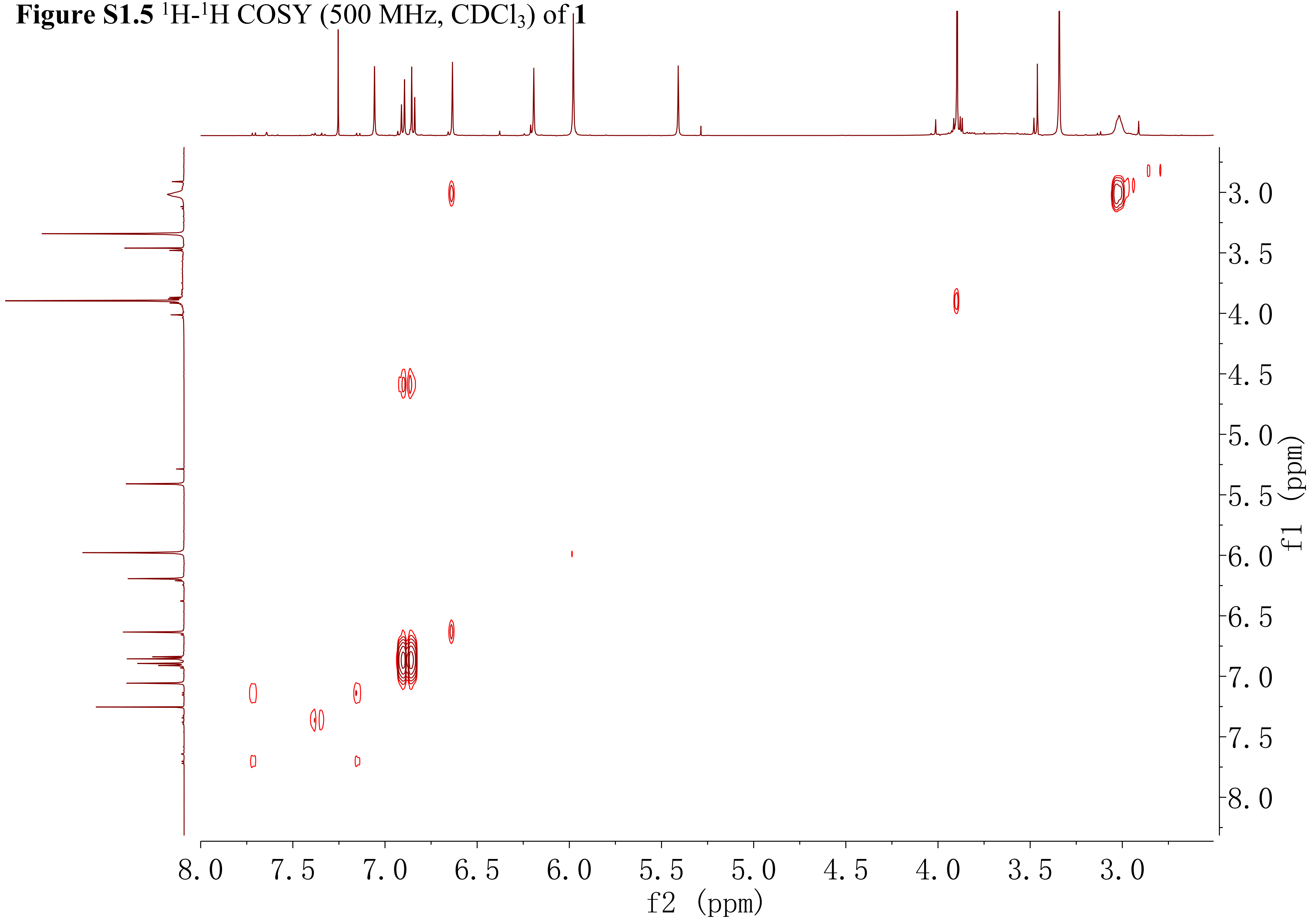

Figure S1.6 HRESIMS spectrum of **1**

Qualitative Analysis Report

**Data Filename**	zwx-37b.d	**Sample Name**	zwx-37b
**Sample Type**	Sample	**Position**	P1-A5
**Instrument Name**	Instrument 1	**User Name**	
**Acq Method**	s.m	**Acquired Time**	4/27/2023 11:51:40 AM
**IRM Calibration Status**	Success	**DA Method**	PCDL.m
**Comment**			

**Sample Group**		**Info.**
**Acquisition SW Version**	6200 series TOF/6500 series Q-TOF B.05.01 (B5125.2)	

User Spectra

Fragmentor Voltage	Collision Energy	Ionization Mode
135	0	ESI

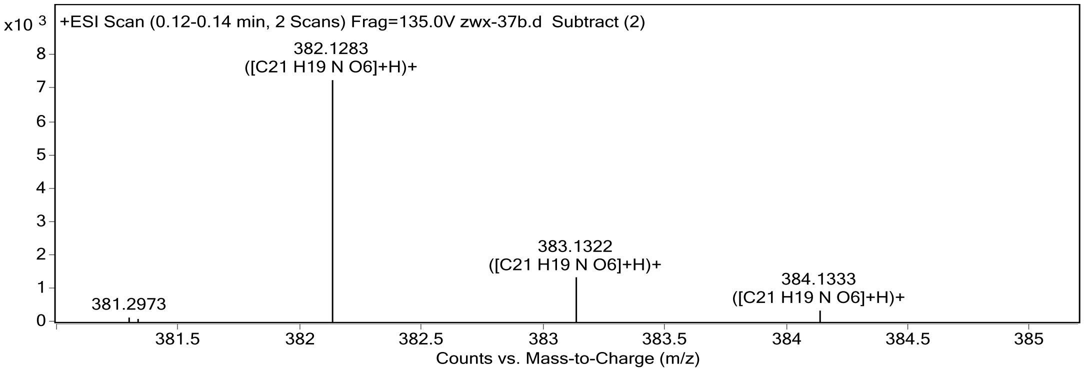

Peak List

m/z	z	Abund	Formula	Ion
97.5297	1	571.88		
221.1554	1	451.72		
274.2738	1	1404.28		
318.3004	1	996.01		
367.1653	1	1694.4		
368.1666	1	539.22		
382.1283	1	7281.47	C21 H19 N O6	(M+H)+
383.1322	1	1369.43	C21 H19 N O6	(M+H)+
415.2128	1	426.21		
637.303	1	484.58		

Formula Calculator Element Limits

Element	Min	Max
C	3	60
H	0	200
O	0	30
N	0	5

Formula Calculator Results

Formula	CalculatedMass	CalculatedMz	Mz	Diff. (mDa)	Diff. (ppm)	DBE
C21 H19 N O6	381.1212	382.1285	382.1283	0.20	0.52	13.0000

--- End Of Report ---

Figure S1.7 IR (KBr disk) spectrum of **1**

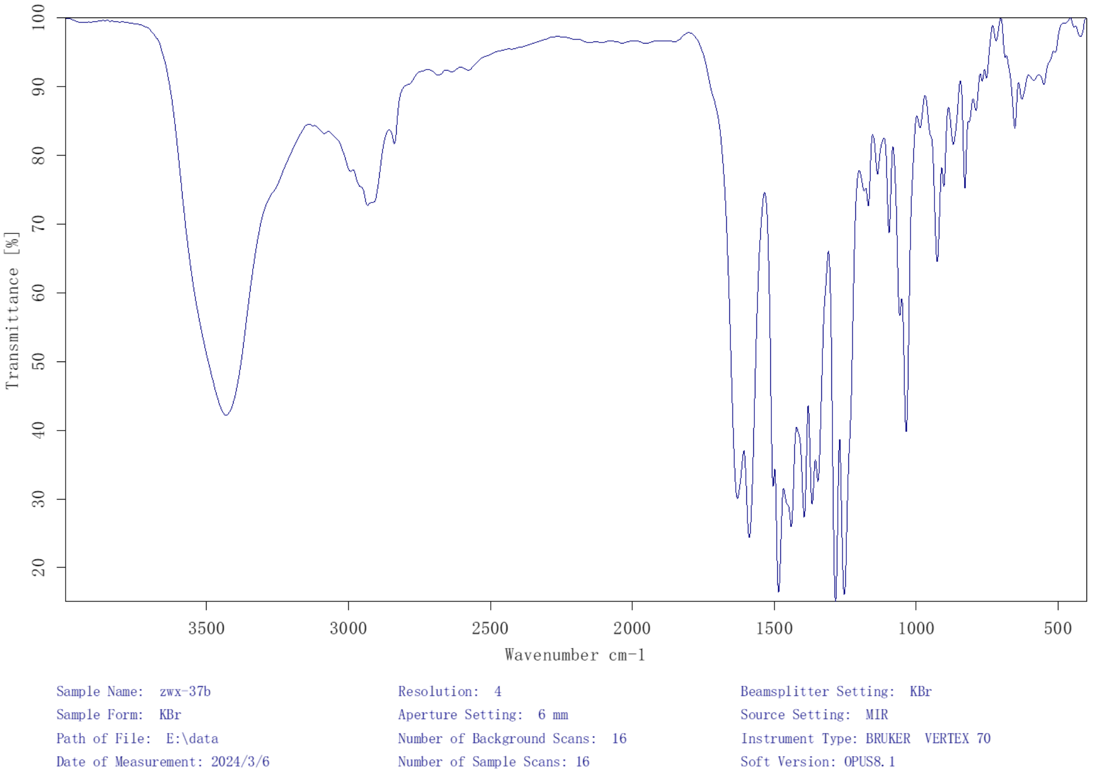

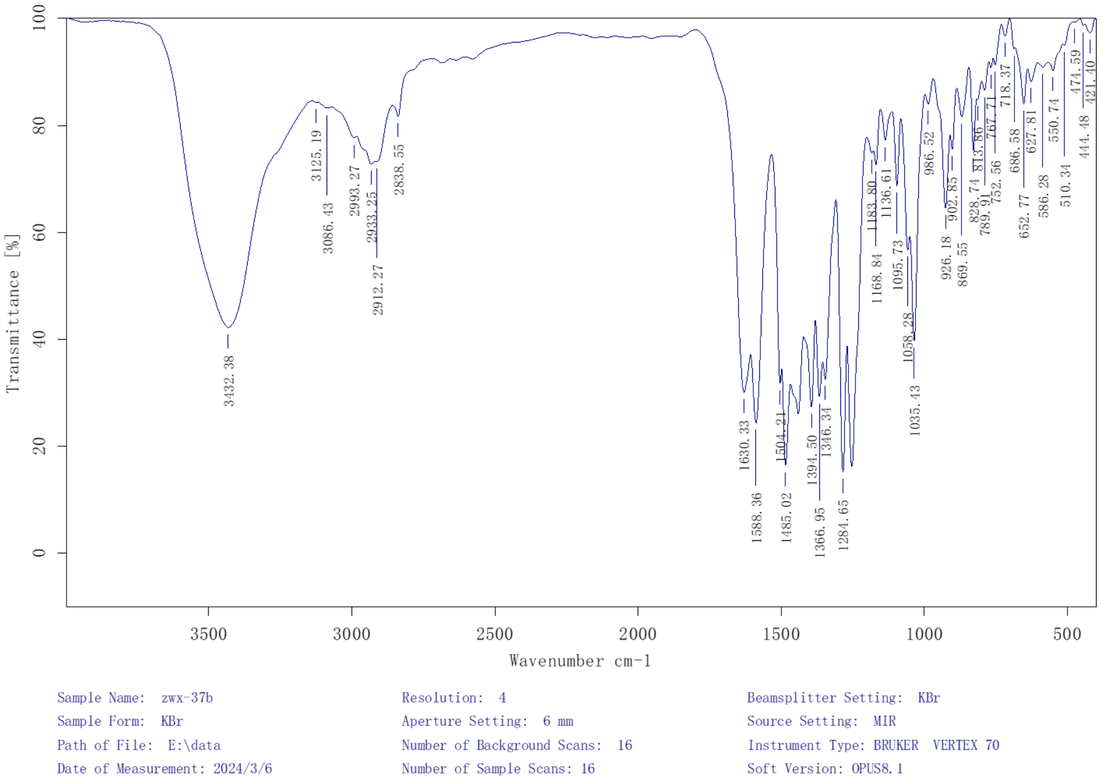

Figure S1.8 X-ray crystal structure of **1**

Crystal data for zwx37b: $C_{21}H_{19}NO_6 \cdot 3(H_2O)$, M = 435.42, a = 40.812(3) Å, b = 13.2173(9) Å, c = 15.3426(9) Å, α = 90°, β = 90.934(7)°, γ = 90°, V = 8275.1(9) Å^3, T = 150.(2) K, space group $C12/c1$, Z = 16, μ(Cu Kα) = 0.931 mm^{-1}, 36414 reflections measured, 7593 independent reflections (R_{int} = 0.3067). The final R_1 values were 0.1195 ($I > 2\sigma(I)$). The final $wR(F^2)$ values were 0.3090 ($I > 2\sigma(I)$). The final R_1 values were 0.1990 (all data). The final $wR(F^2)$ values were 0.3769 (all data). The goodness of fit on F^2 was 0.996.

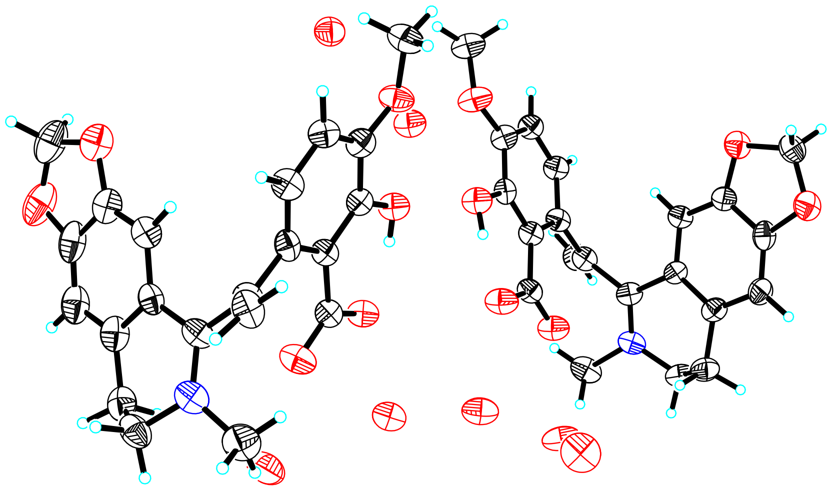

View of the molecules in an asymmetric unit.

Displacement ellipsoids are drawn at the 30% probability level.

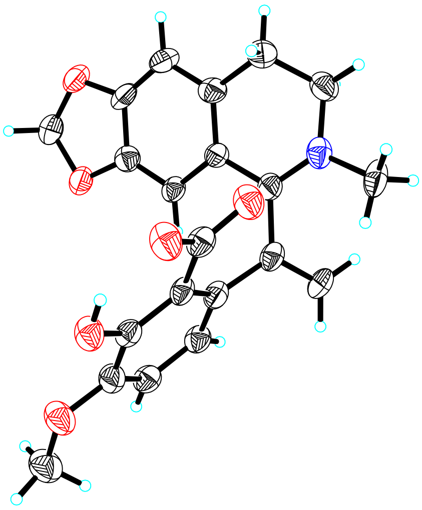

View of a molecule of zwx37b.

Displacement ellipsoids are drawn at the 30% probability level.

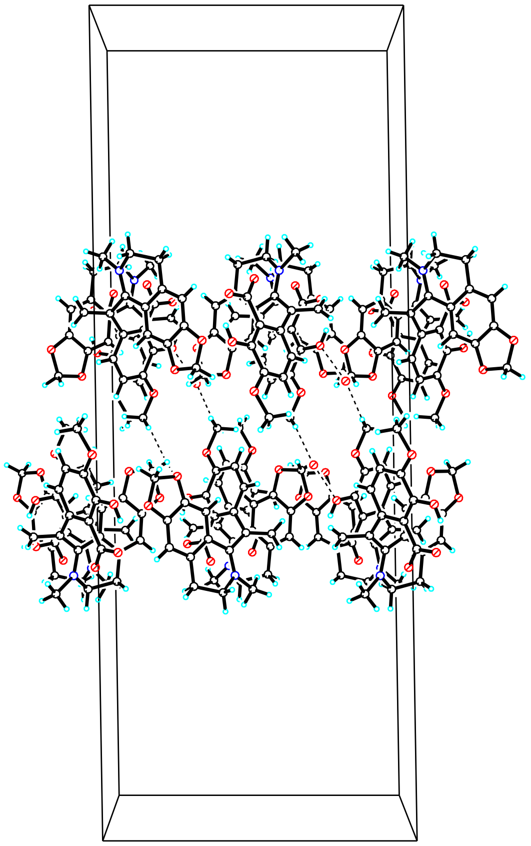

View of the pack drawing of zwx37b.

Hydrogen-bonds are shown as dashed lines.

Table 1. Crystal data and structure refinement for zwx37b_0m.

Identification code	global	
Empirical formula	C21 H25 N O9	
Formula weight	435.42	
Temperature	150(2) K	
Wavelength	1.54178 Å	
Crystal system	Monoclinic	
Space group	C 1 2/c 1	
Unit cell dimensions	a = 40.812(3) Å	α= 90°.
	b = 13.2173(9) Å	β= 90.934(7)°.

	c = 15.3426(9) Å	γ = 90°.
Volume	8275.1(9) $Å^3$	
Z	16	
Density (calculated)	1.398 Mg/m^3	
Absorption coefficient	0.931 mm^{-1}	
F(000)	3680	
Crystal size	0.160 x 0.150 x 0.020 mm^3	
Theta range for data collection	2.17 to 69.06°.	
Index ranges	-49<=h<=49, -15<=k<=15, -18<=l<=17	
Reflections collected	36414	
Independent reflections	7593 [R(int) = 0.3067]	
Completeness to theta = 69.06°	98.6 %	
Absorption correction	Semi-empirical from equivalents	
Max. and min. transmission	0.98 and 0.47	
Refinement method	Full-matrix least-squares on F^2	
Data / restraints / parameters	7593 / 7 / 571	
Goodness-of-fit on F^2	0.996	
Final R indices [I>2sigma(I)]	R1 = 0.1195, wR2 = 0.3090	
R indices (all data)	R1 = 0.1990, wR2 = 0.3769	
Extinction coefficient	0.00068(12)	
Largest diff. peak and hole	0.466 and -0.478 e.$Å^{-3}$	

Figure S2.1 ^{1}H NMR (500 MHz, $CDCl_3$) of **2**

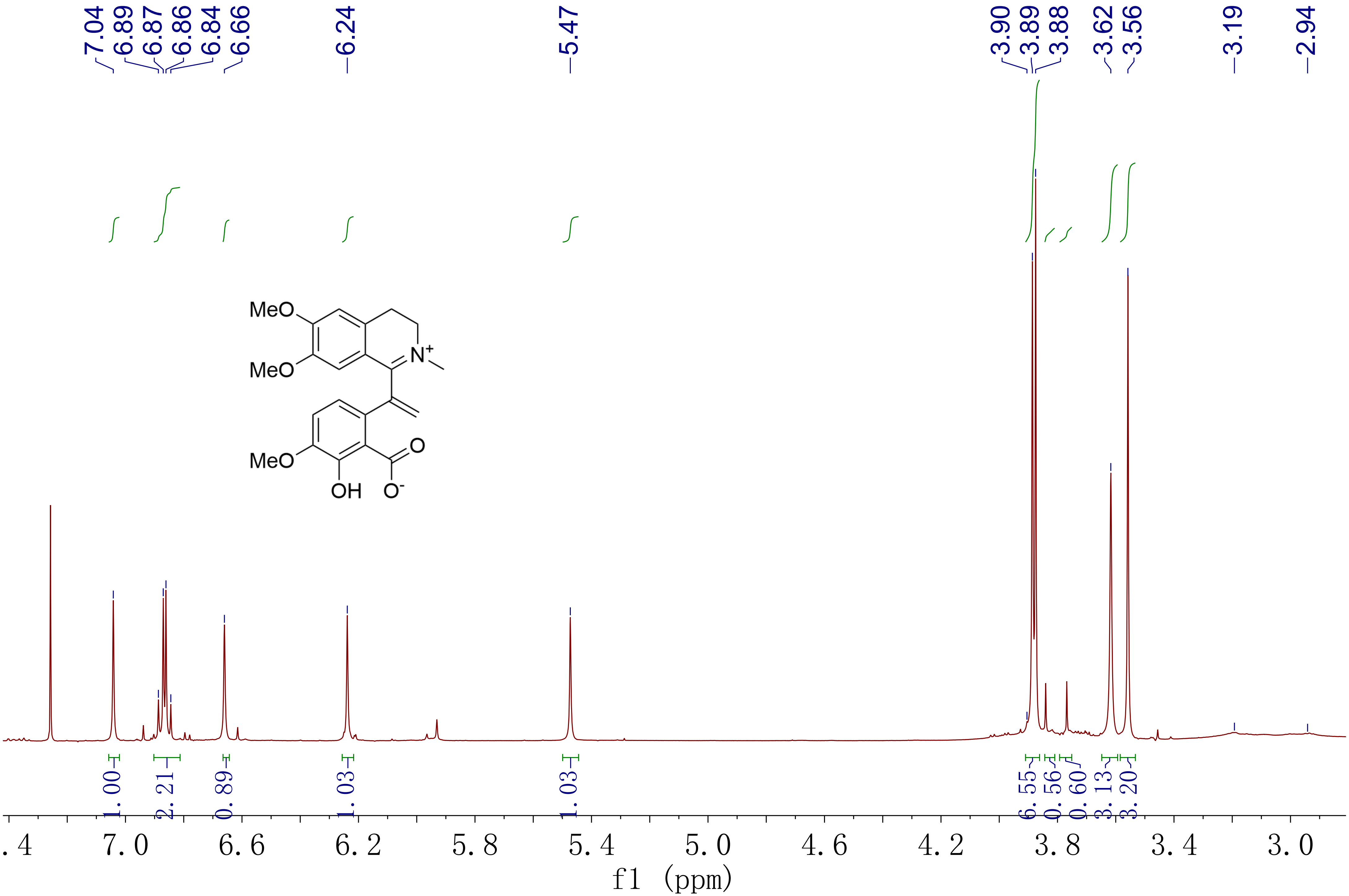

Figure S2.2 ^{13}C NMR (125 MHz, $CDCl_3$) of **2**

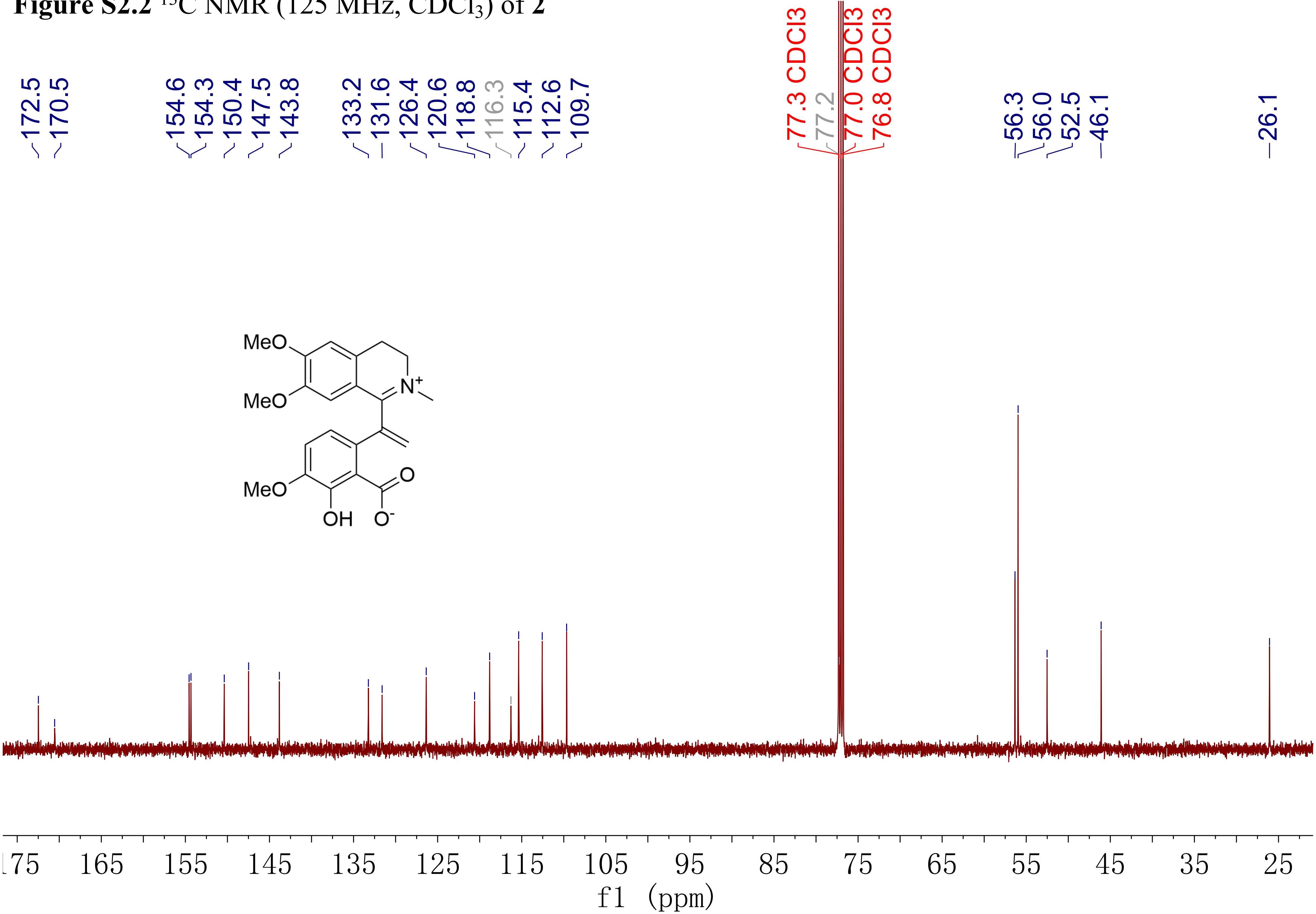

Figure S2.3 HSQC (500 MHz, $CDCl_3$) of **2**

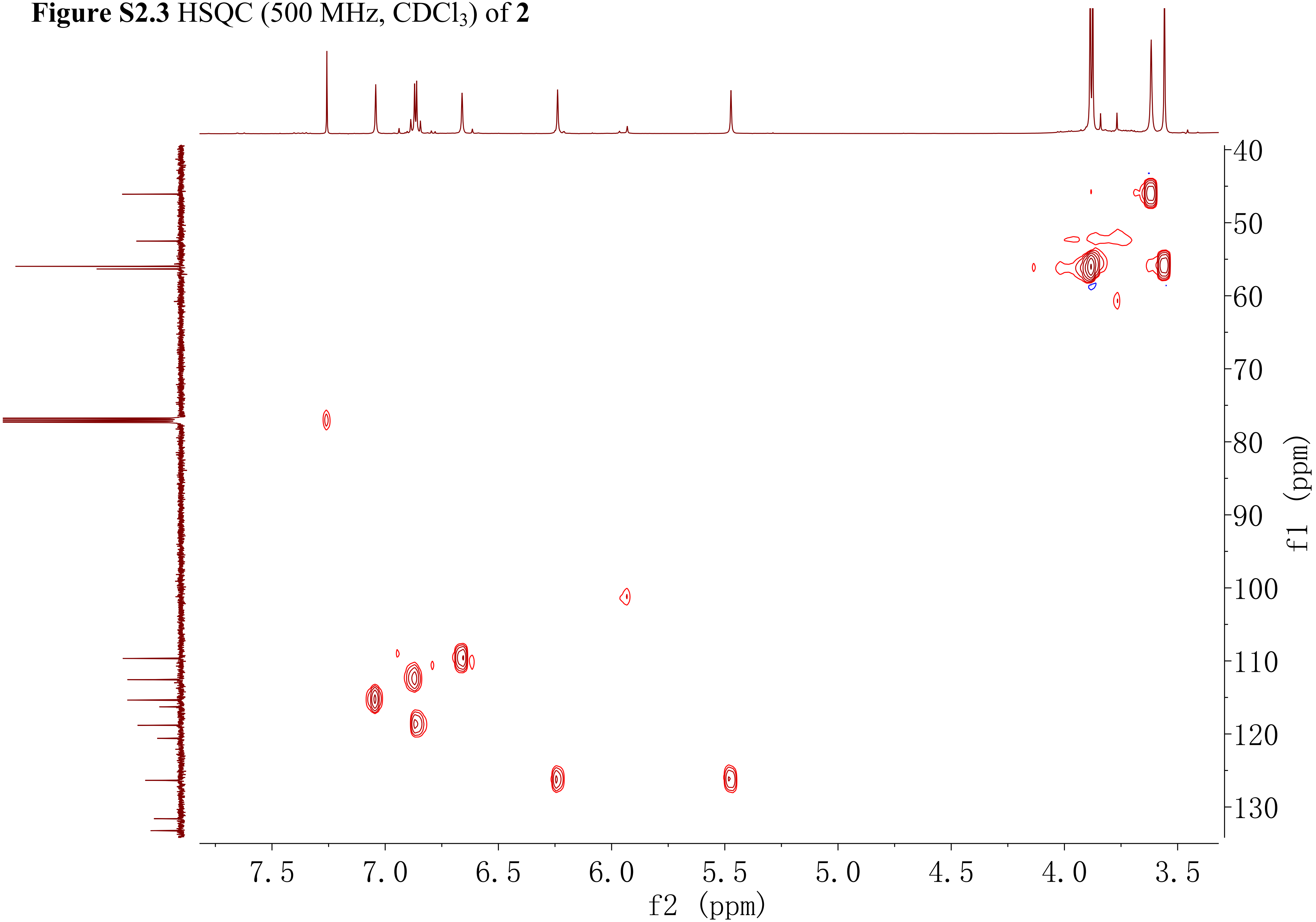

Figure S2.4 HMBC (500 MHz, $CDCl_3$) of **2**

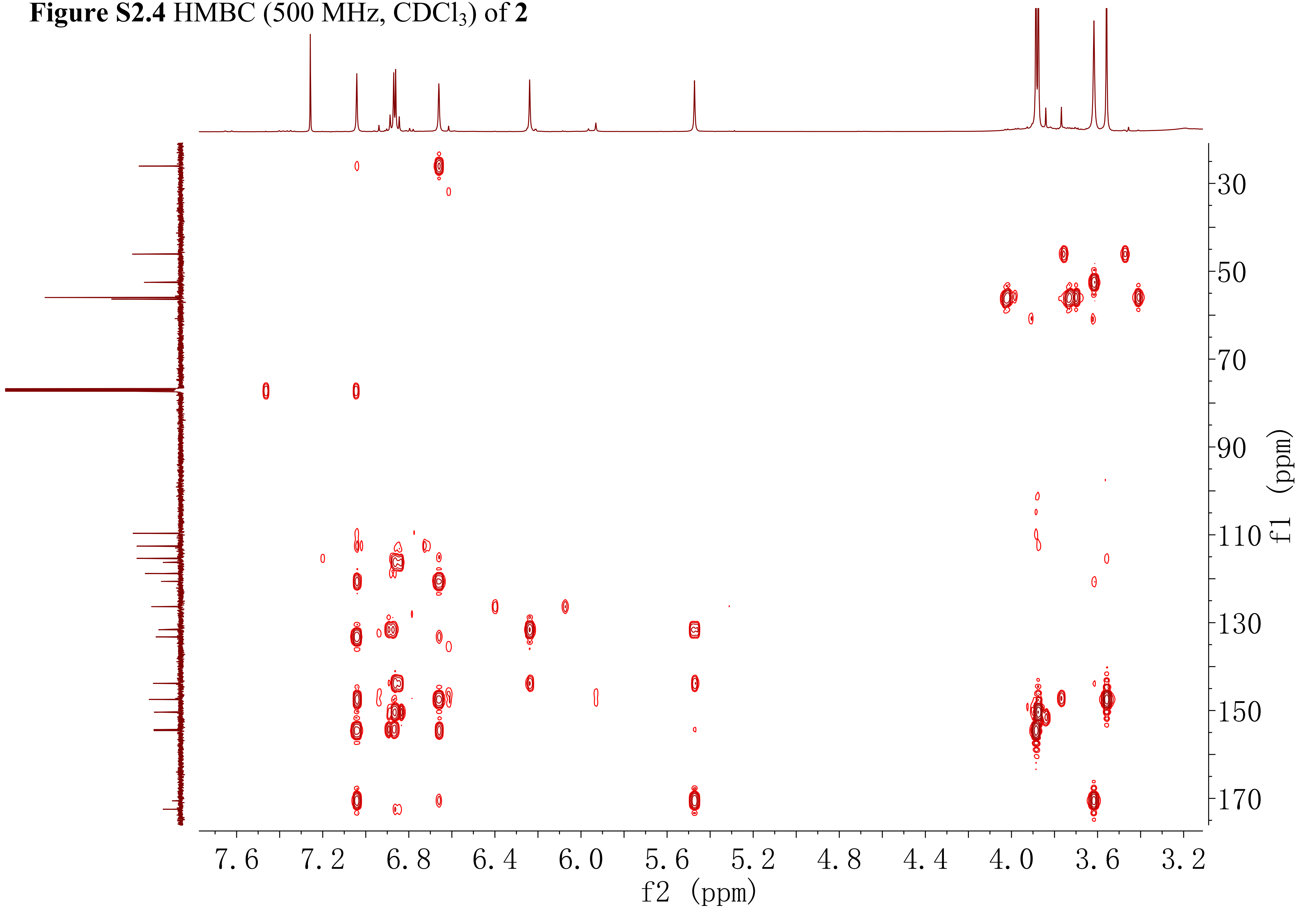

Figure S2.5 ^{1}H-^{1}H COSY (500 MHz, $CDCl_3$) of **2**

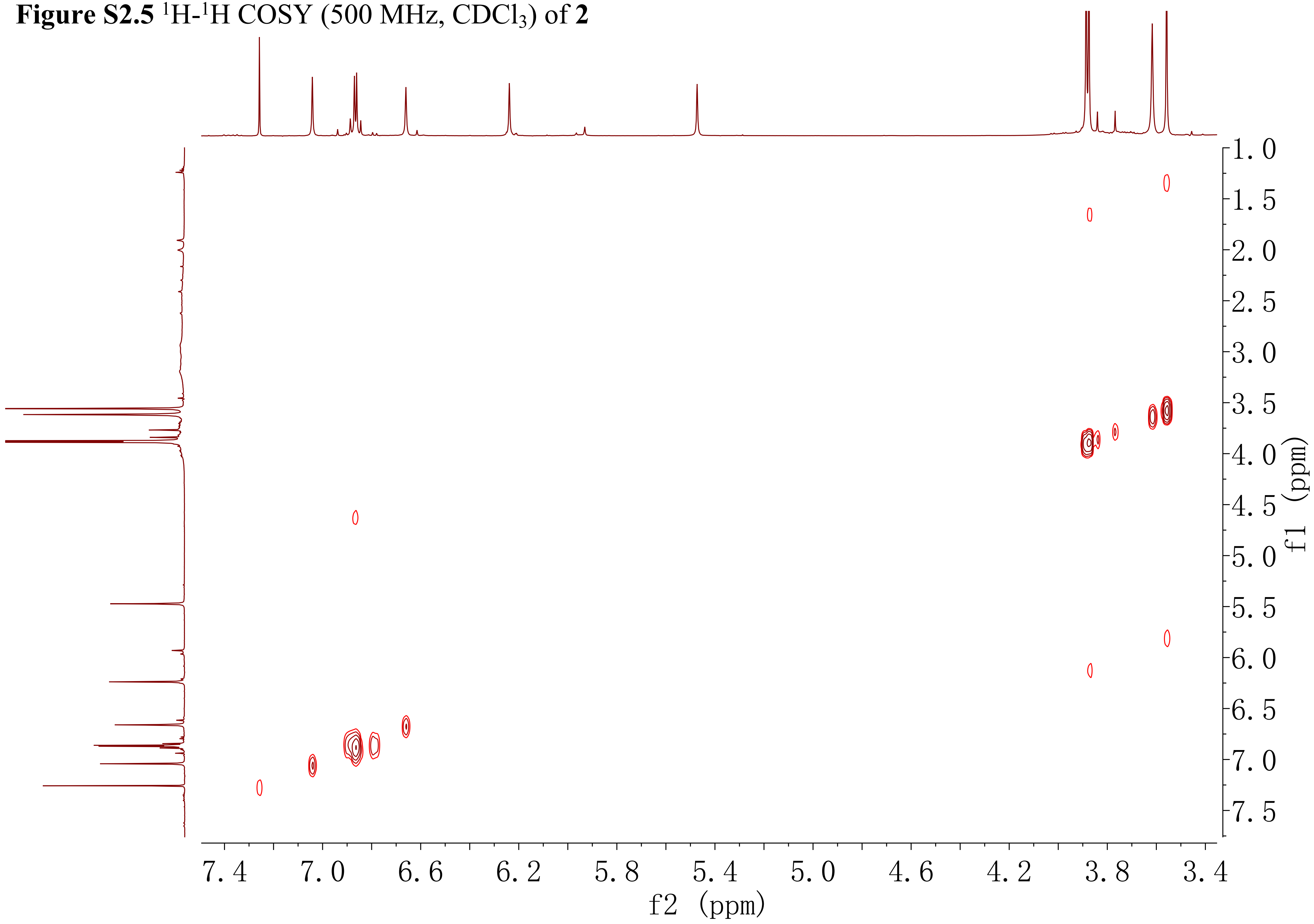

Figure S2.6 HRESIMS spectrum of **2**

Qualitative Analysis Report

**Data Filename**	zwx-44.d	**Sample Name**	zwx-44
**Sample Type**	Sample	**Position**	P1-A1
**Instrument Name**	Instrument 1	**User Name**	
**Acq Method**	s.m	**Acquired Time**	6/13/2023 3:15:05 PM
**IRM Calibration Status**	Success	**DA Method**	PCDL.m
**Comment**			

Sample Group **Info.**

Acquisition SW Version 6200 series TOF/6500 series Q-TOF B.05.01 (B5125.2)

User Spectra

Fragmentor Voltage	Collision Energy	Ionization Mode
135	0	ESI

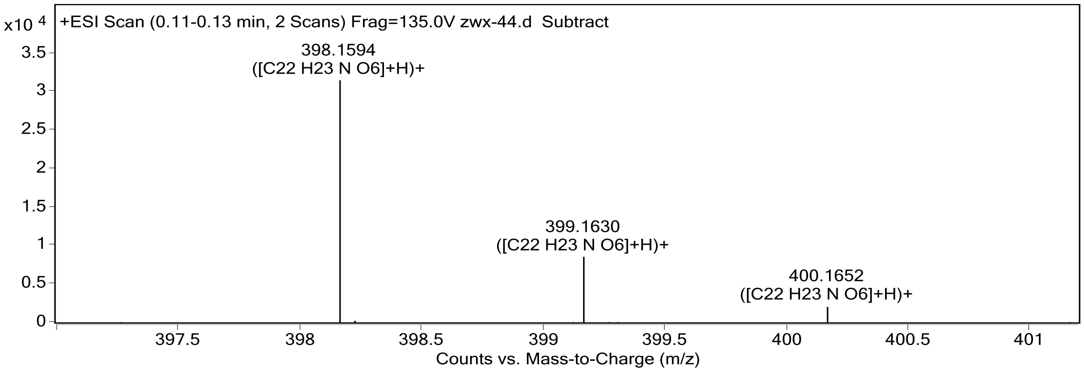

Peak List

m/z	z	Abund	Formula	Ion
367.1659	1	2709.92		
370.1648	1	18241.05		
371.1698	1	3831.49		
398.1594	1	31512.21	C22 H23 N O6	(M+H)+
399.163	1	8559.05	C22 H23 N O6	(M+H)+
400.1652	1	2169.02	C22 H23 N O6	(M+H)+
420.1414	1	18400.02		
421.1445	1	3529.2		
488.1306	1	1707.21		
923.0127	1	1782.49		

Formula Calculator Element Limits

Element	Min	Max
C	3	60
H	0	120
O	0	30
N	0	3

Formula Calculator Results

Formula	CalculatedMass	CalculatedMz	Mz	Diff. (mDa)	Diff. (ppm)	DBE
C22 H23 N O6	397.1525	398.1598	398.1594	0.40	1.00	12.0000

--- End Of Report ---

Figure S2.7 IR (KBr disk) spectrum of **2**

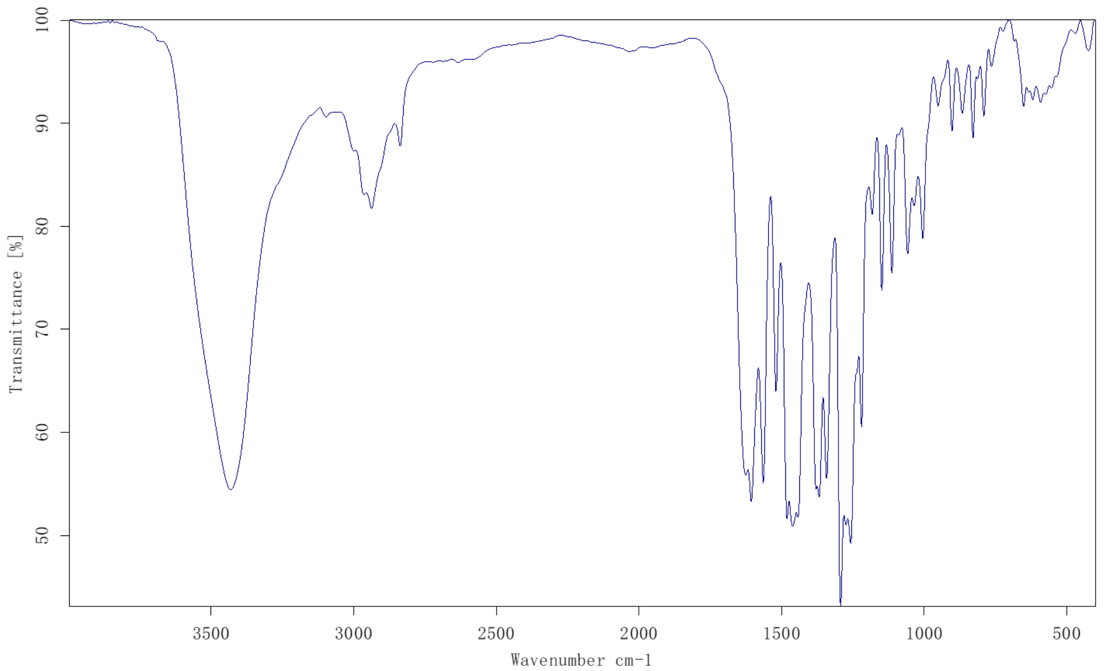

Sample Name: zwx-44
Sample Form: KBr
Path of File: E:\data
Date of Measurement: 2024/3/6

Resolution: 4
Aperture Setting: 6 mm
Number of Background Scans: 16
Number of Sample Scans: 16

Beamsplitter Setting: KBr
Source Setting: MIR
Instrument Type: BRUKER VERTEX 70
Soft Version: OPUS8.1

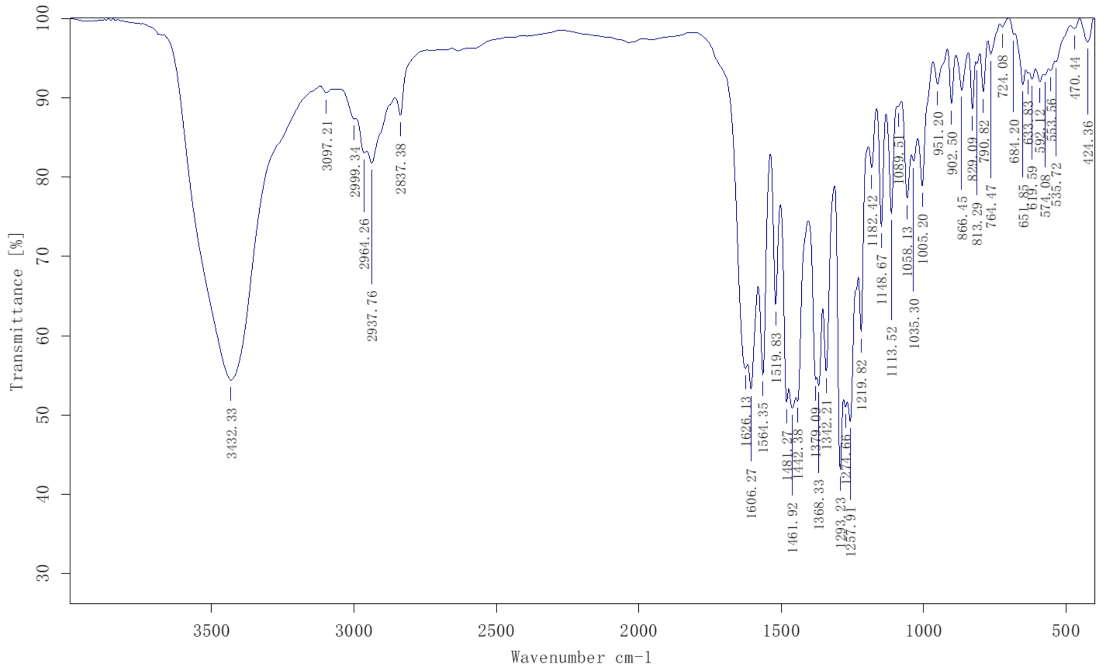

Sample Name: zwx-44
Sample Form: KBr
Path of File: E:\data
Date of Measurement: 2024/3/6

Resolution: 4
Aperture Setting: 6 mm
Number of Background Scans: 16
Number of Sample Scans: 16

Beamsplitter Setting: KBr
Source Setting: MIR
Instrument Type: BRUKER VERTEX 70
Soft Version: OPUS8.1

Figure S3.1 ^{1}H NMR (500 MHz, $CDCl_3$) of **3**

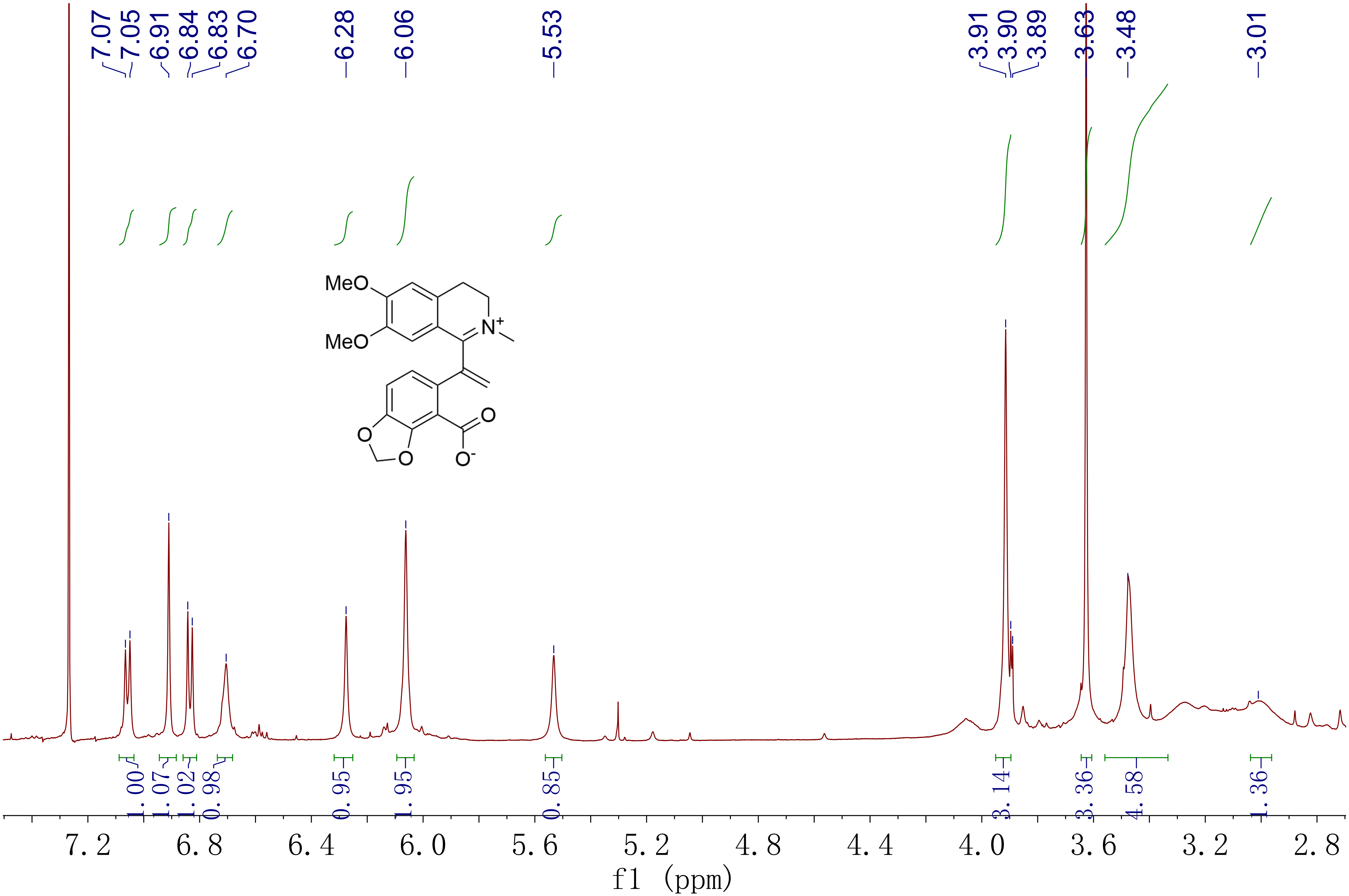

Figure S3.2 ^{13}C NMR (125 MHz, $CDCl_3$) of **3**

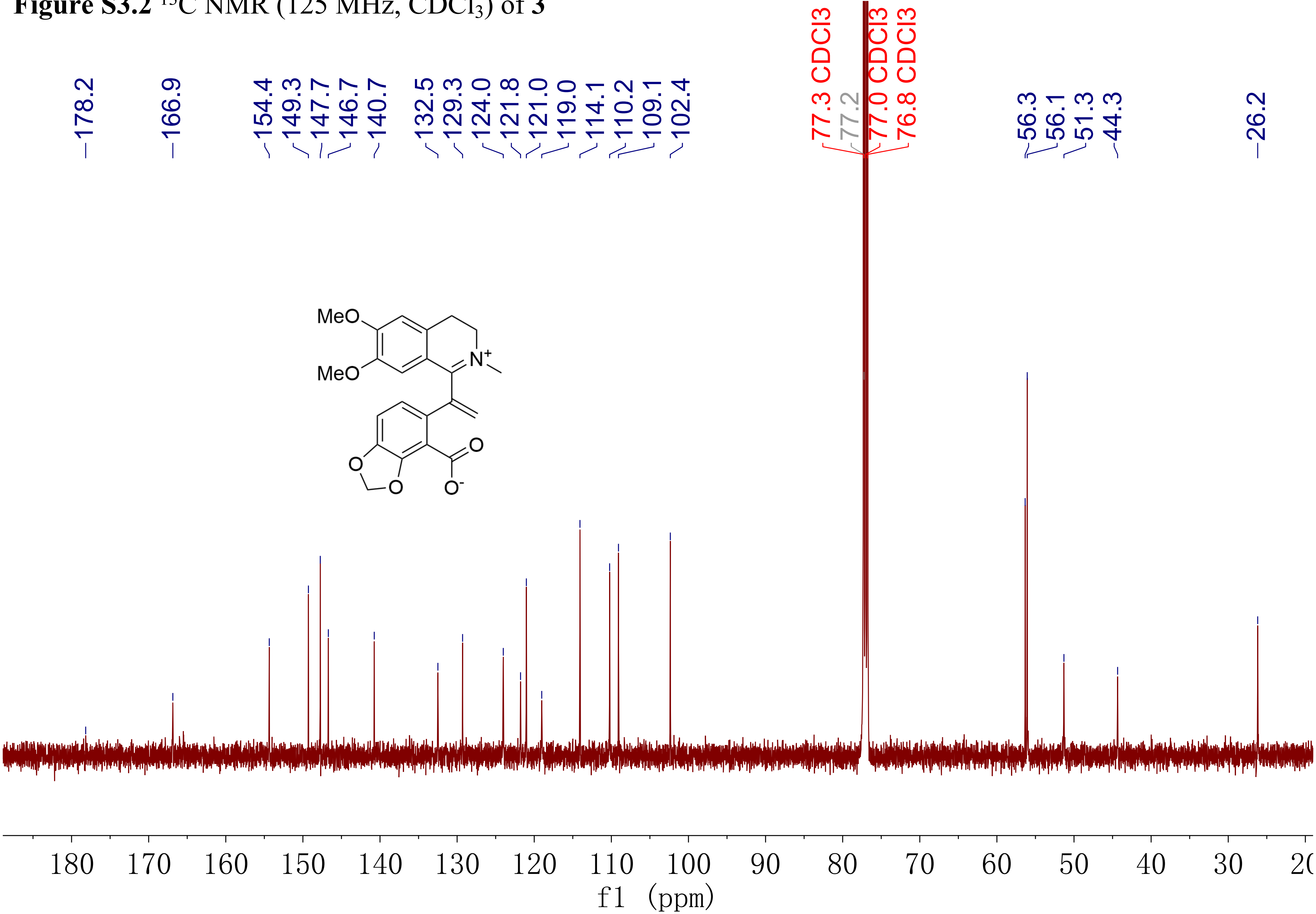

Figure S3.3 HSQC (500 MHz, $CDCl_3$) of **3**

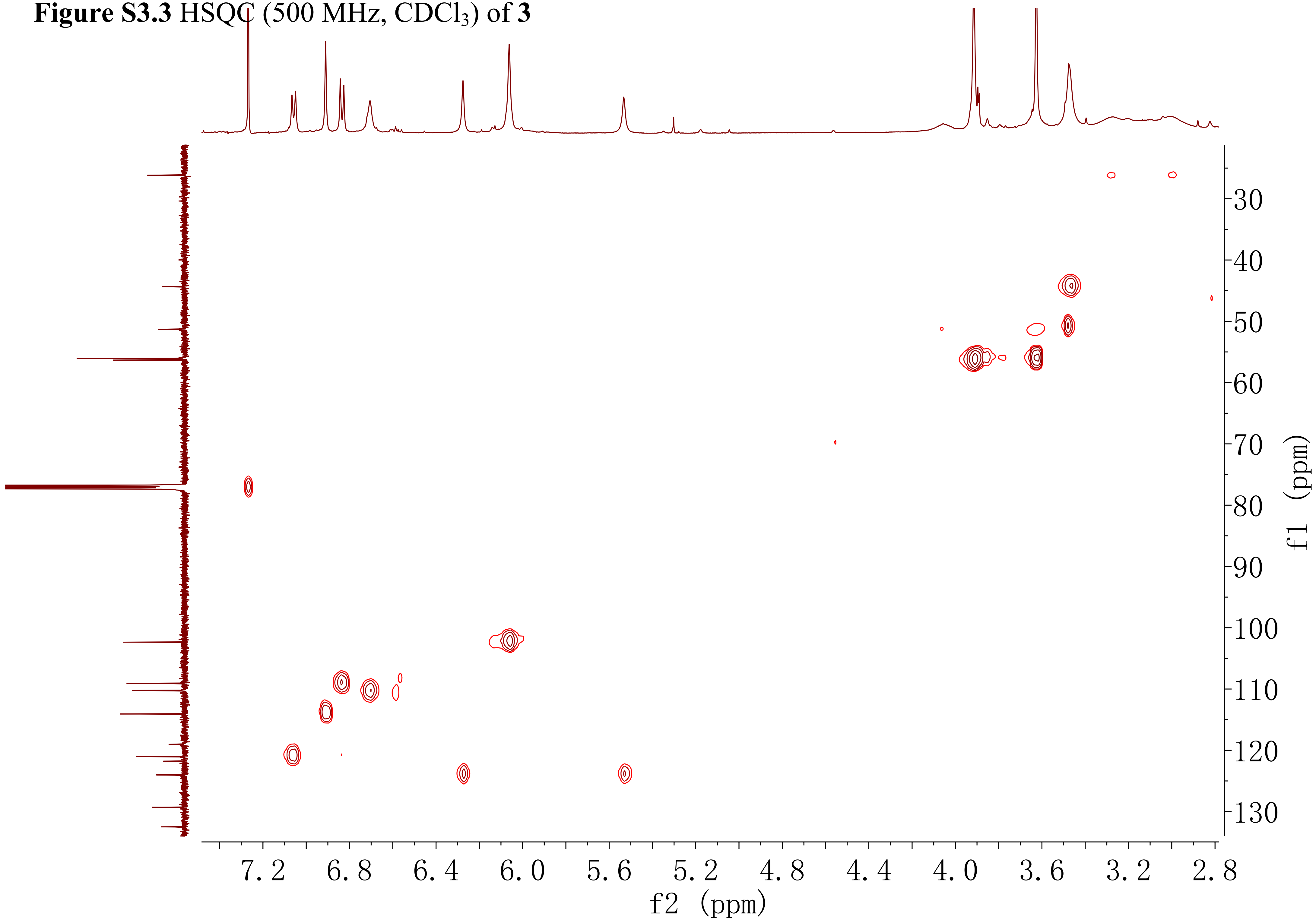

Figure S3.4 HMBC (500 MHz, $CDCl_3$) of **3**

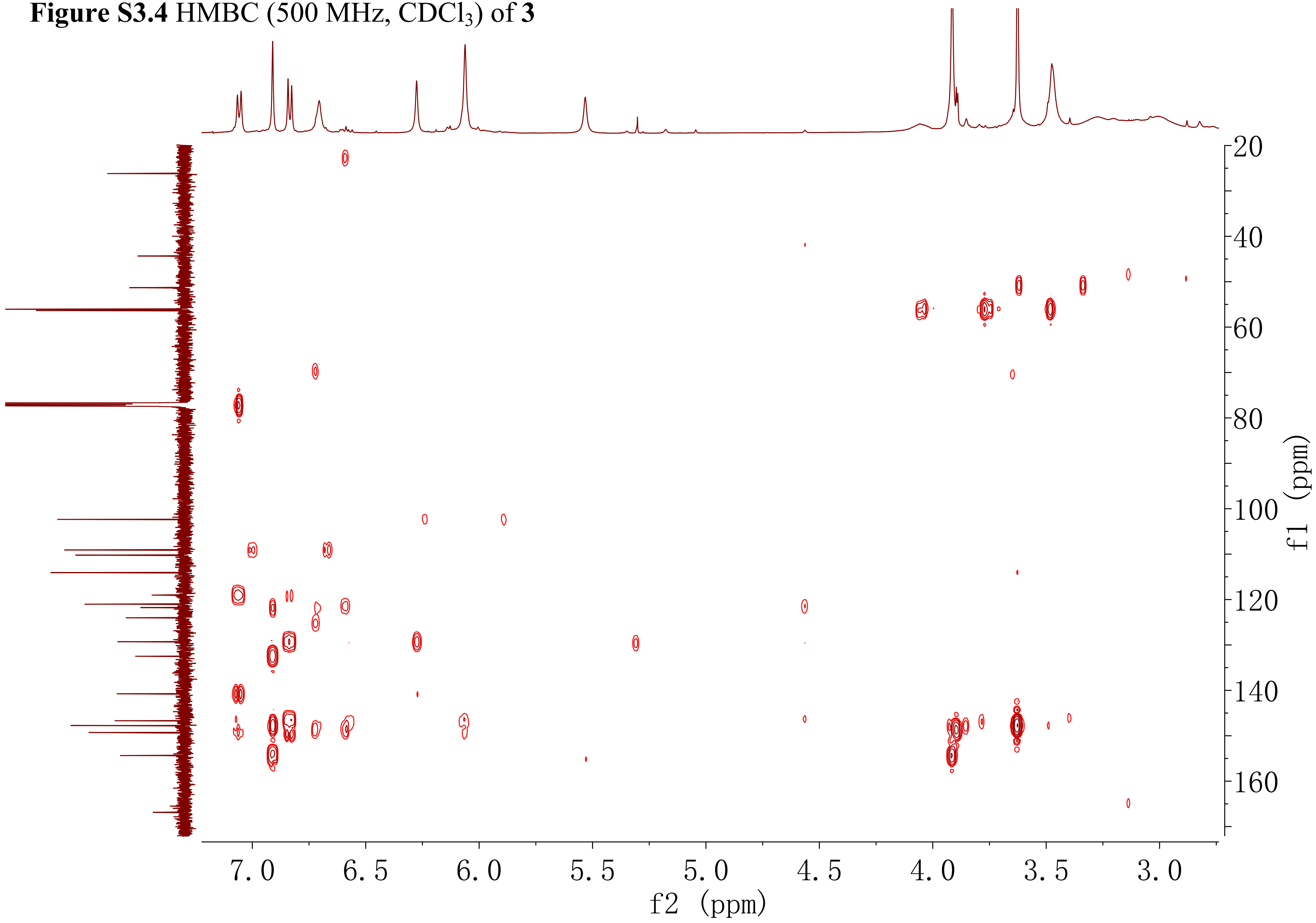

Figure S3.5 ^1H-^1H COSY (500 MHz, $CDCl_3$) of **3**

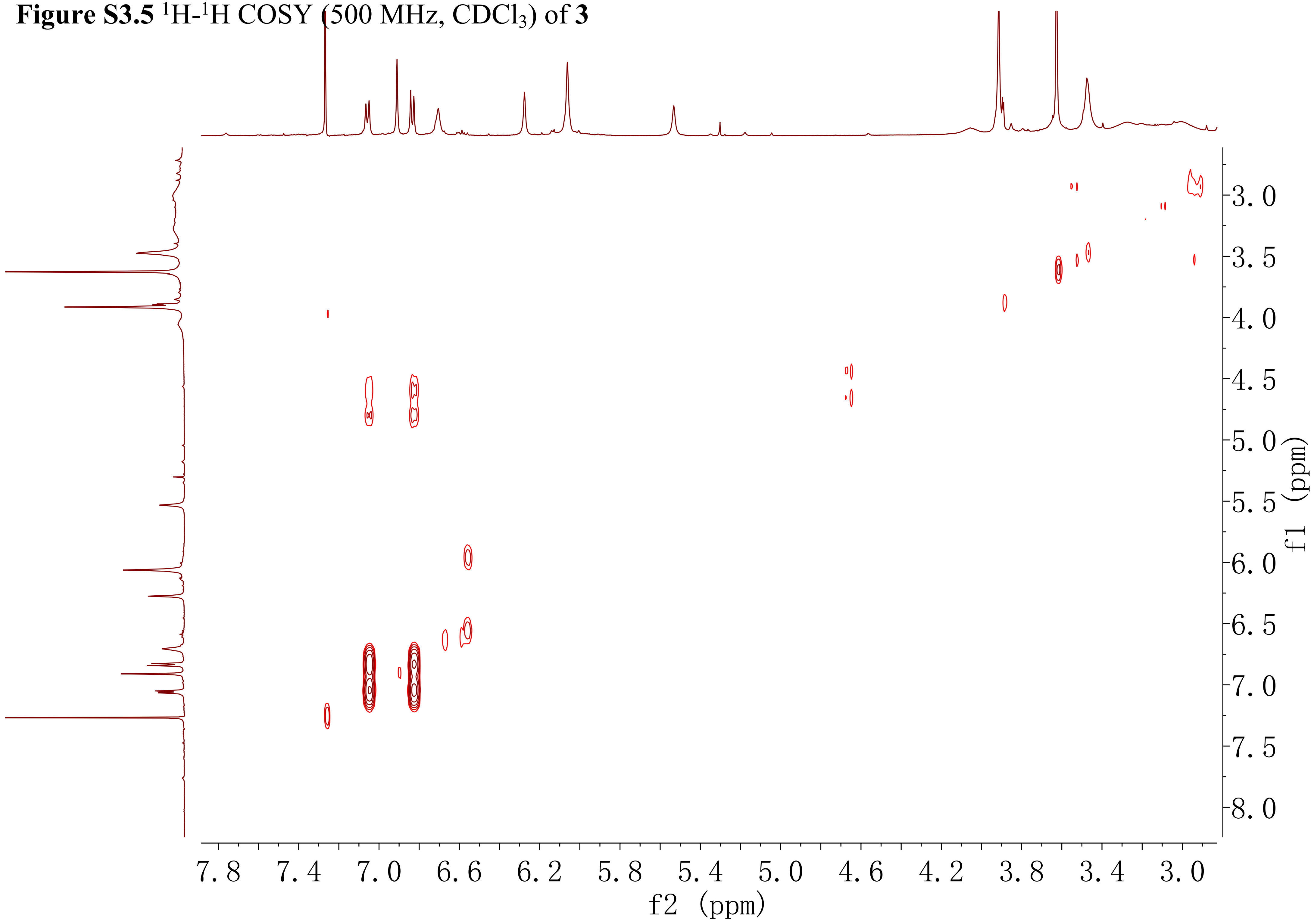

Figure S3.6 HRESIMS spectrum of **3**

Qualitative Analysis Report

**Data Filename**	zwh-14.d	**Sample Name**	zwh-14
**Sample Type**	Sample	**Position**	P1-B6
**Instrument Name**	Instrument 1	**User Name**	
**Acq Method**	s.m	**Acquired Time**	9/15/2023 1:34:52 PM
**IRM Calibration Status**	Success	**DA Method**	PCDL.m
**Comment**			

Sample Group **Info.**
Acquisition SW Version 6200 series TOF/6500 series Q-TOF B.05.01 (B5125.2)

User Spectra

Fragmentor Voltage	Collision Energy	Ionization Mode
135	0	ESI

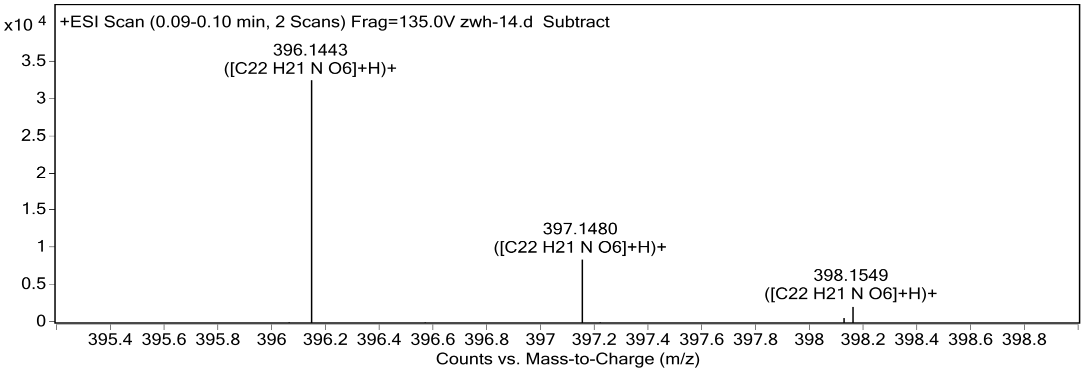

Peak List

m/z	z	Abund	Formula	Ion
109.9435	1	1153.92		
122.9639	1	1800.45		
125.9863	1	1047.41		
141.9583	1	1194.67		
384.1442	1	38955.43		
385.1479	1	8214.41		
386.1502	1	1548.04		
396.1443	1	32559.11	C22 H21 N O6	(M+H)+
397.148	1	8534.03	C22 H21 N O6	(M+H)+
398.1549	1	2296.51	C22 H21 N O6	(M+H)+

Formula Calculator Element Limits

Element	Min	Max
C	3	60
H	0	150
O	0	20
N	0	5

Formula Calculator Results

Formula	CalculatedMass	CalculatedMz	Mz	Diff. (mDa)	Diff. (ppm)	DBE
C22 H21 N O6	395.1369	396.1442	396.1443	-0.10	-0.25	13.0000

--- End Of Report ---

Figure S3.7 IR (KBr disk) spectrum of **3**

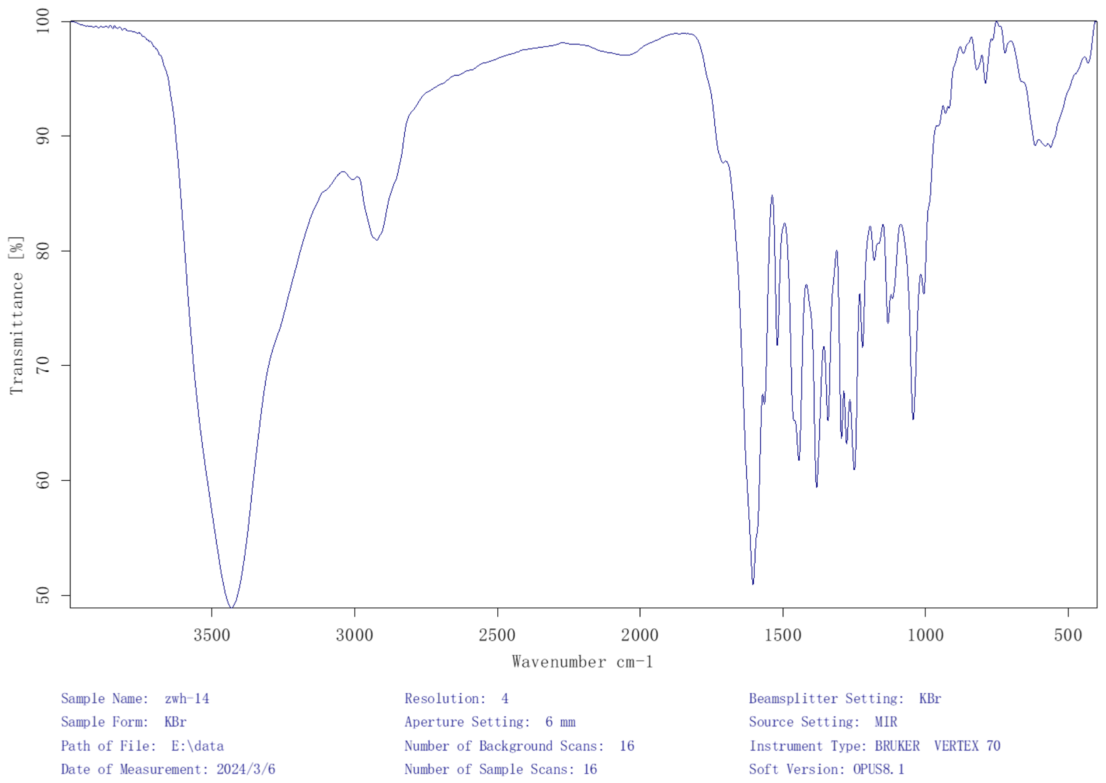

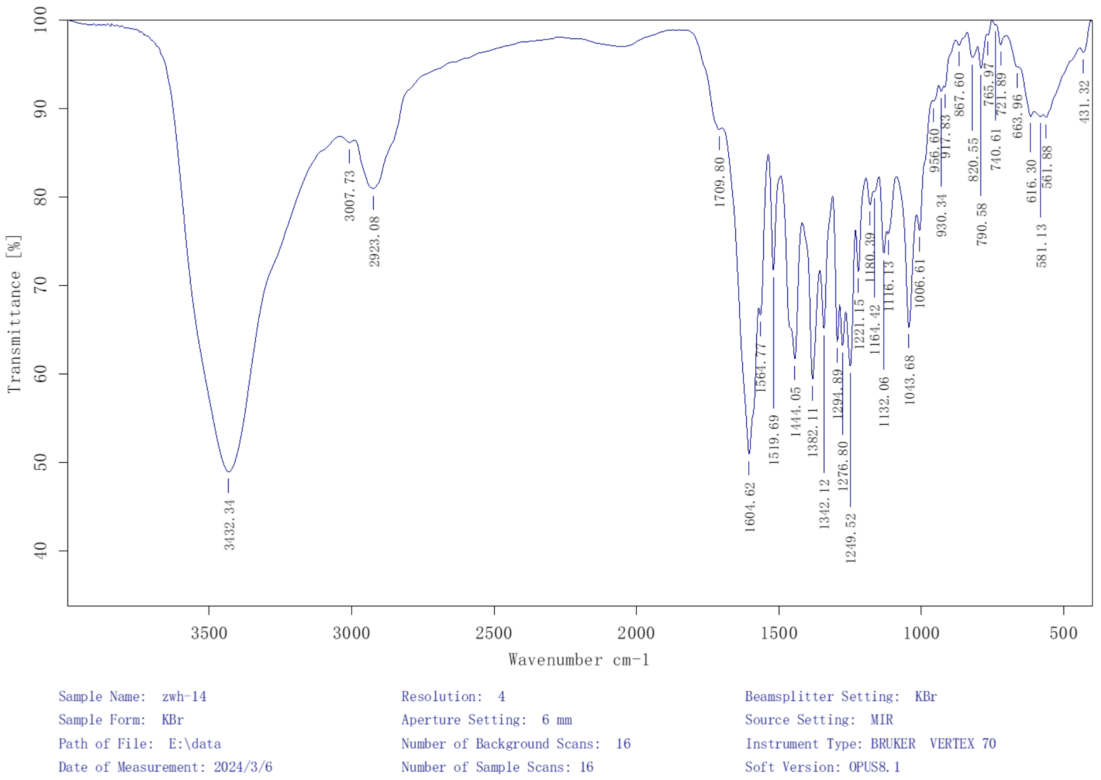

Figure S4.1 ^{1}H NMR (500 MHz, $CDCl_3$) of **4**

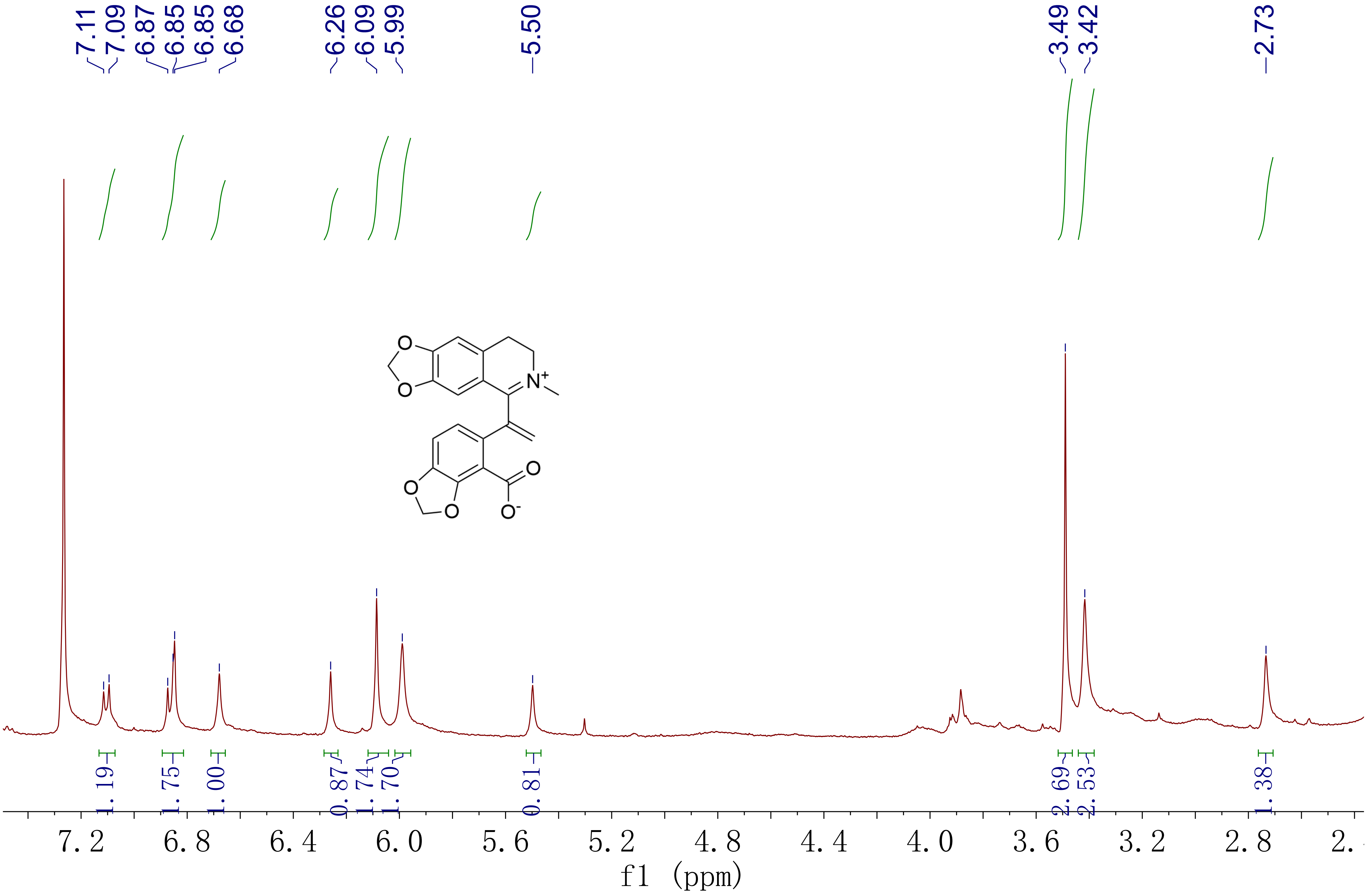

Figure S4.2 ^{13}C NMR (125 MHz, $CDCl_3$) of **4**

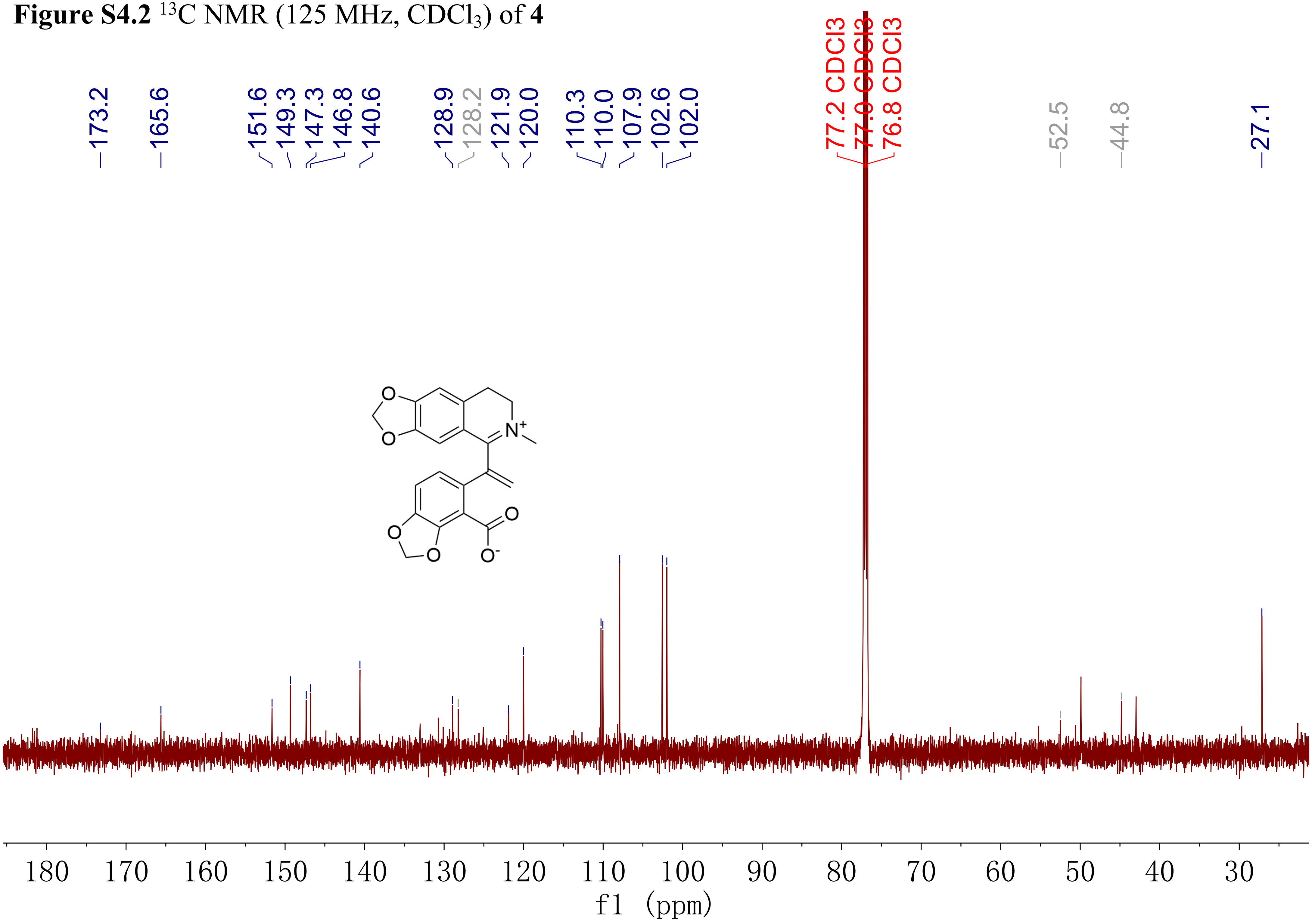

Figure S4.3 HSQC (500 MHz, $CDCl_3$) of **4**

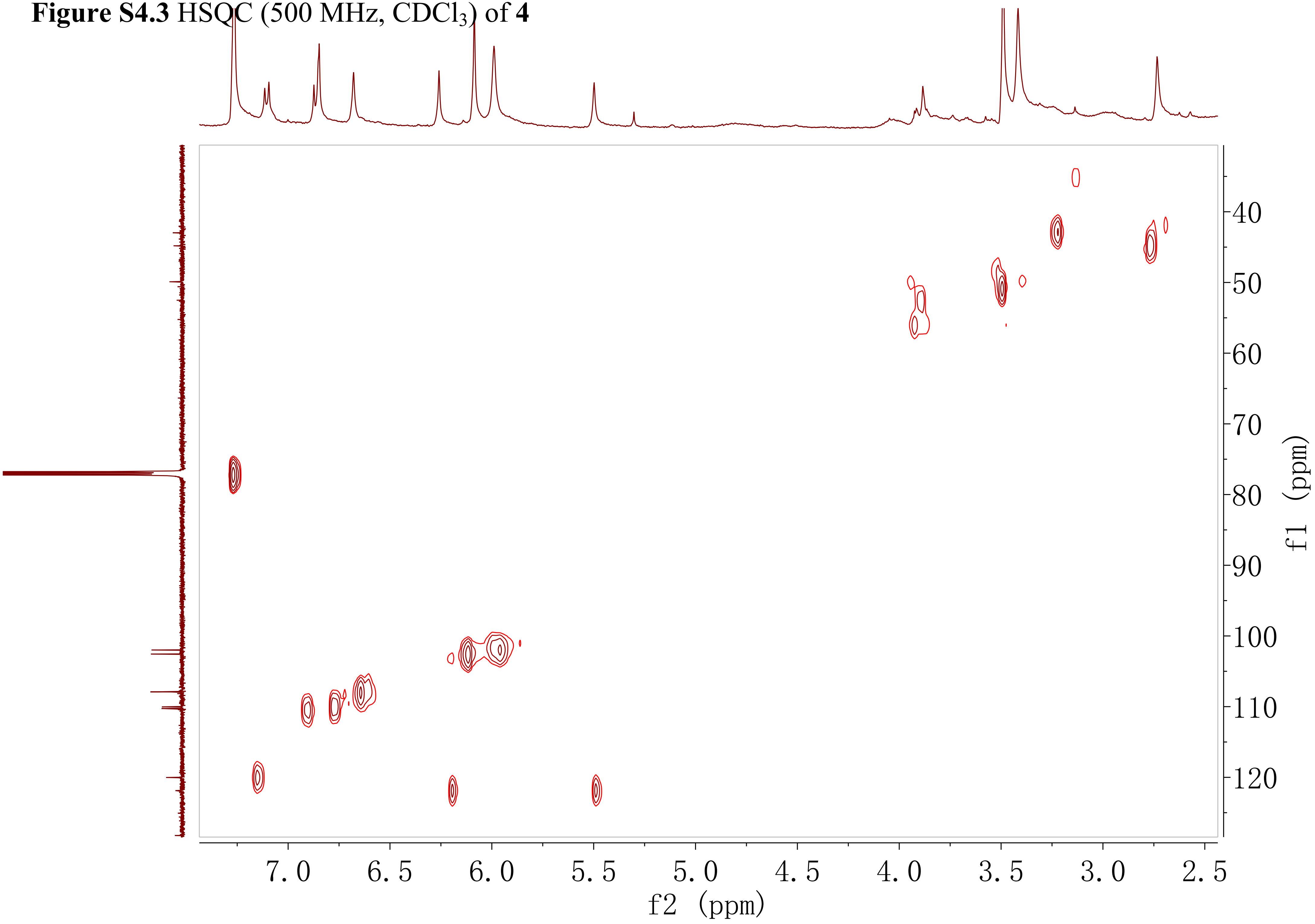

Figure S4.4 HMBC (500 MHz, $CDCl_3$) of **4**

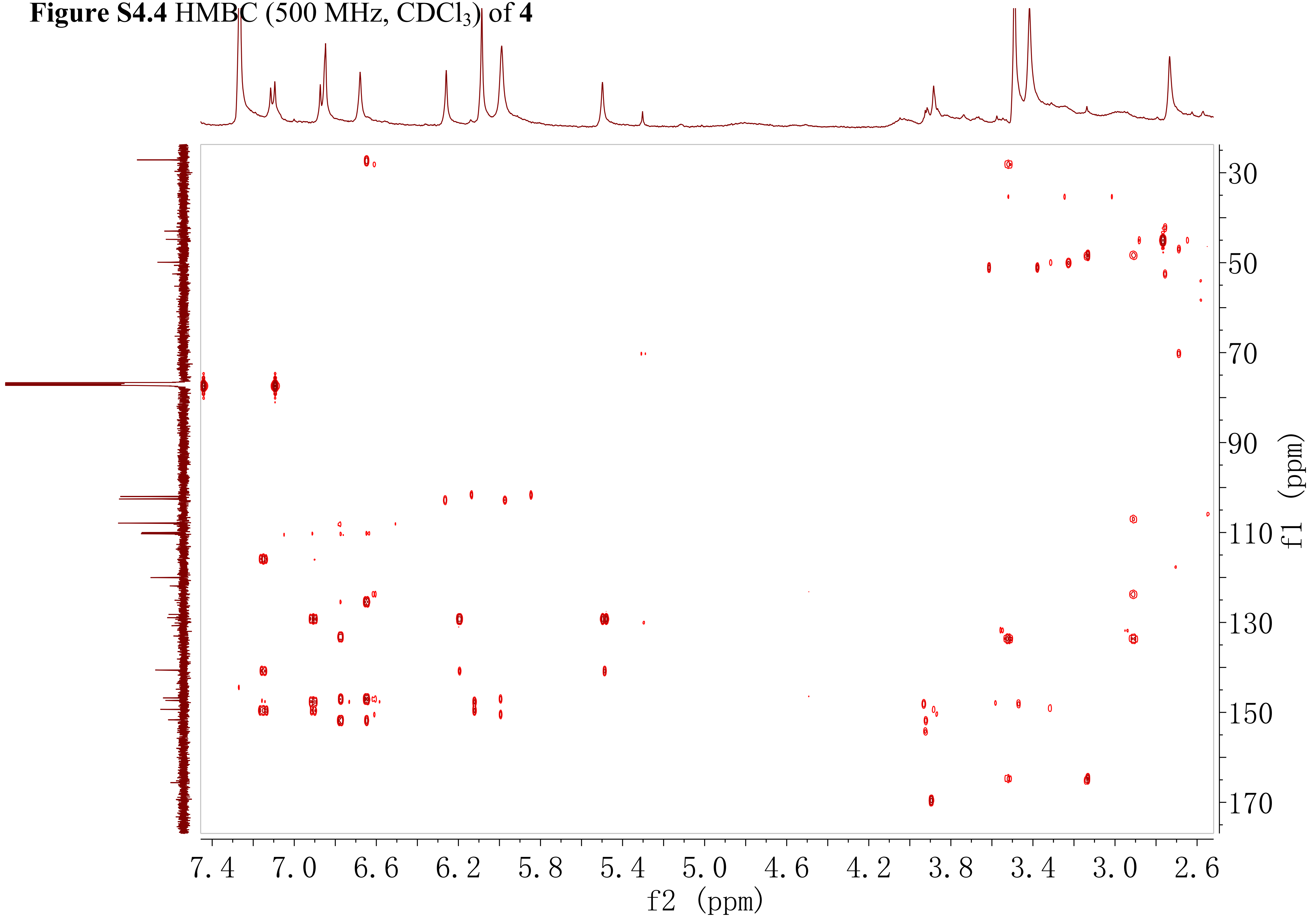

Figure S4.5 ^{1}H-^{1}H COSY (500 MHz, $CDCl_3$) of **4**

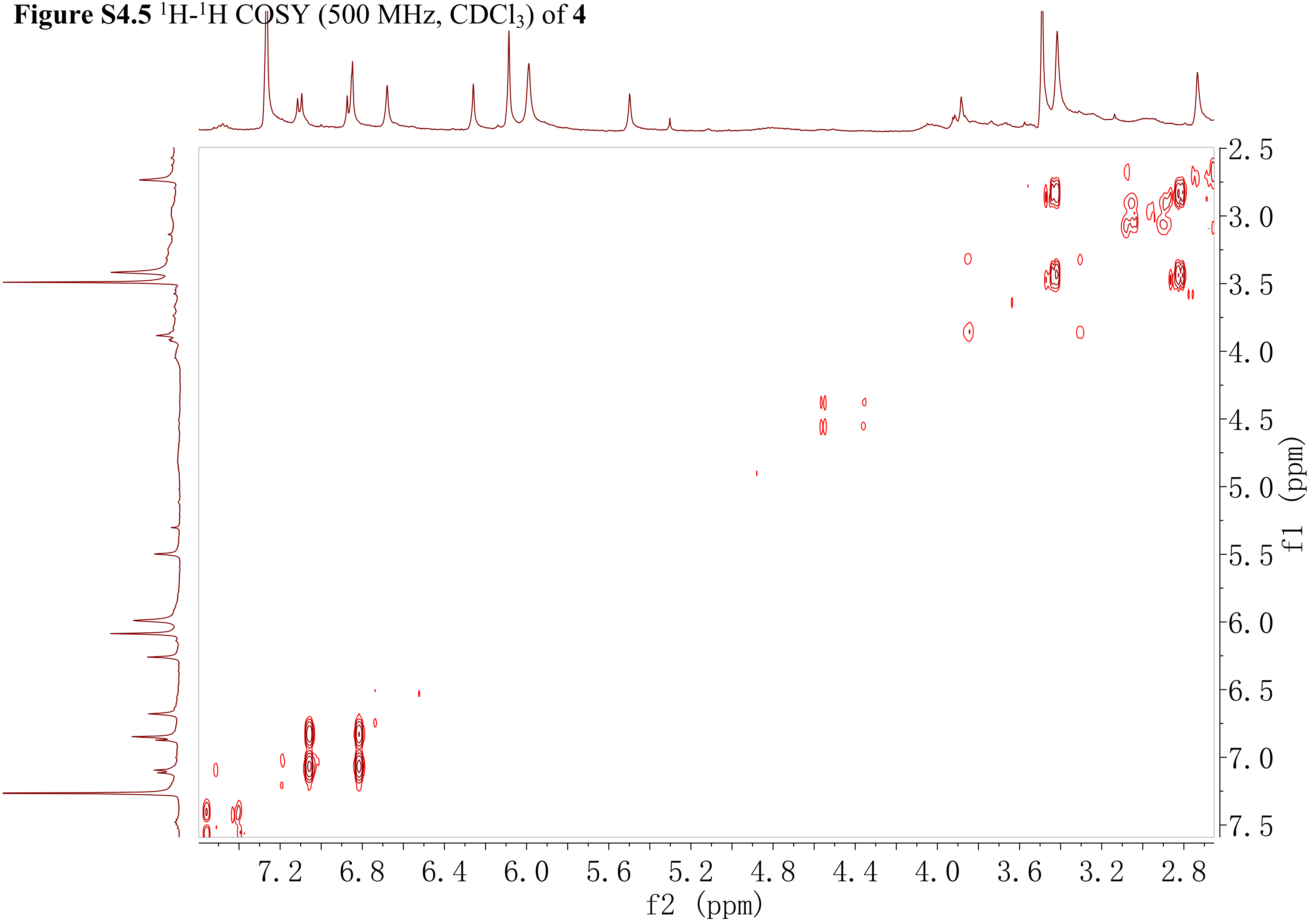

Figure S4.6 HRESIMS spectrum of **4**

Qualitative Analysis Report

**Data Filename**	zwh-5f.d	**Sample Name**	zwh-5f
**Sample Type**	Sample	**Position**	P1-F4
**Instrument Name**	Instrument 1	**User Name**	
**Acq Method**	s.m	**Acquired Time**	3/5/2024 11:02:27 AM
**IRM Calibration Status**	Success	**DA Method**	PCDL.m
**Comment**			

**Sample Group**		**Info.**
**Acquisition SW Version**	6200 series TOF/6500 series Q-TOF B.05.01 (B5125.2)	

User Spectra

Fragmentor Voltage	Collision Energy	Ionization Mode
135	0	ESI

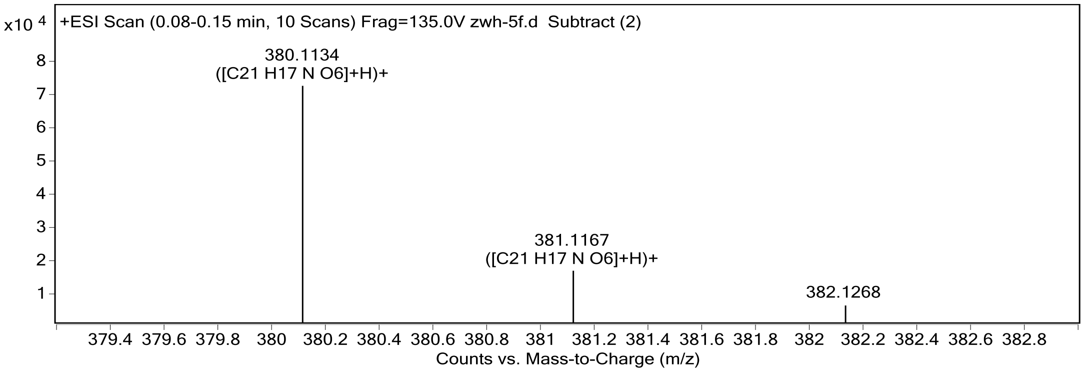

Peak List

m/z	z	Abund	Formula	Ion
103.9559	1	2724.68		
136.9315	1	2608.03		
144.9821	1	7719.68		
146.9804	1	3024.5		
368.1132	1	4400.36		
380.1134	1	73137.76	C21 H17 N O6	(M+H)+
381.1167	1	17241.98	C21 H17 N O6	(M+H)+
382.1268	1	7089.29		
453.3111	1	2821.32		
613.4834	1	2425.43		

Formula Calculator Element Limits

Element	Min	Max
C	3	120
H	0	250
O	0	50
N	0	3

Formula Calculator Results

Formula	CalculatedMass	CalculatedMz	Mz	Diff. (mDa)	Diff. (ppm)	DBE
C21 H17 N O6	379.1056	380.1129	380.1134	-0.50	-1.32	14.0000

--- End Of Report ---

Figure S4.7 IR (KBr disk) spectrum of **4**

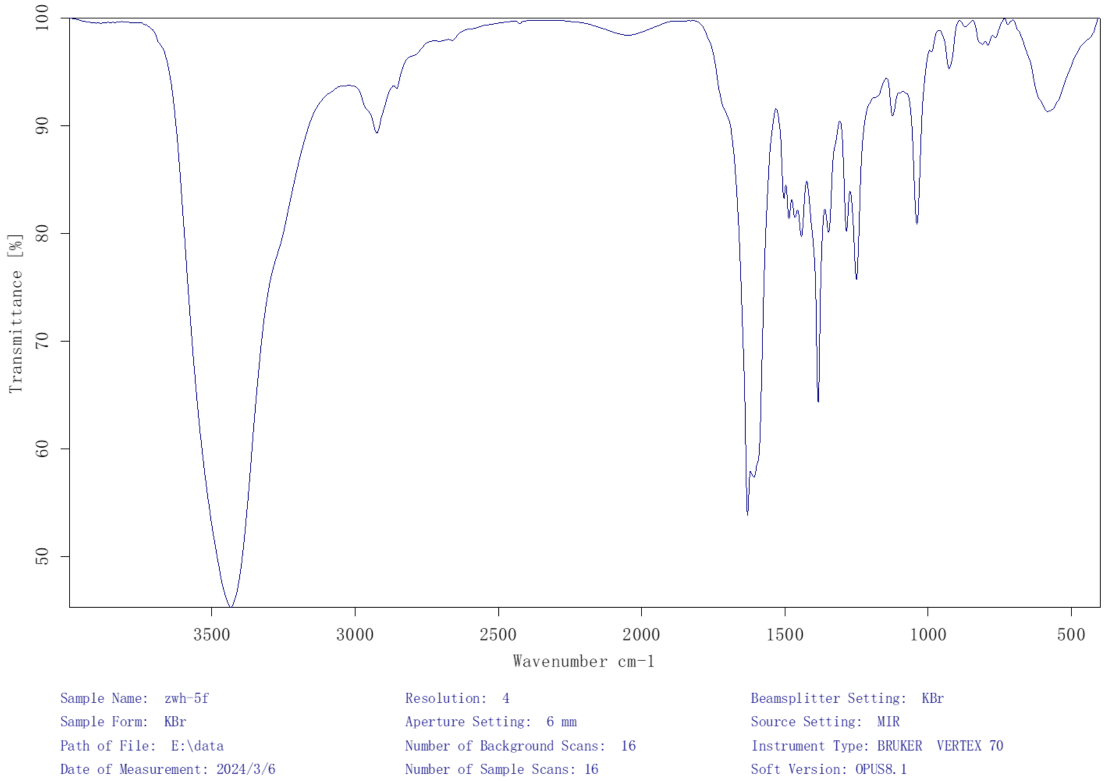

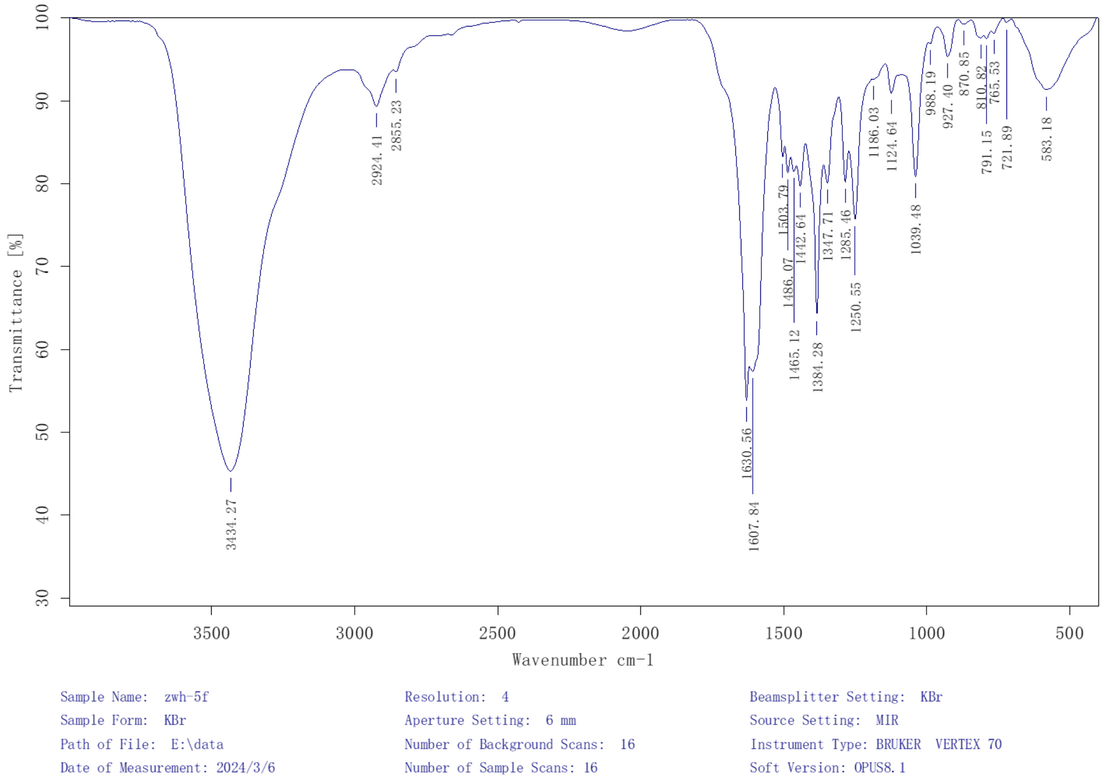

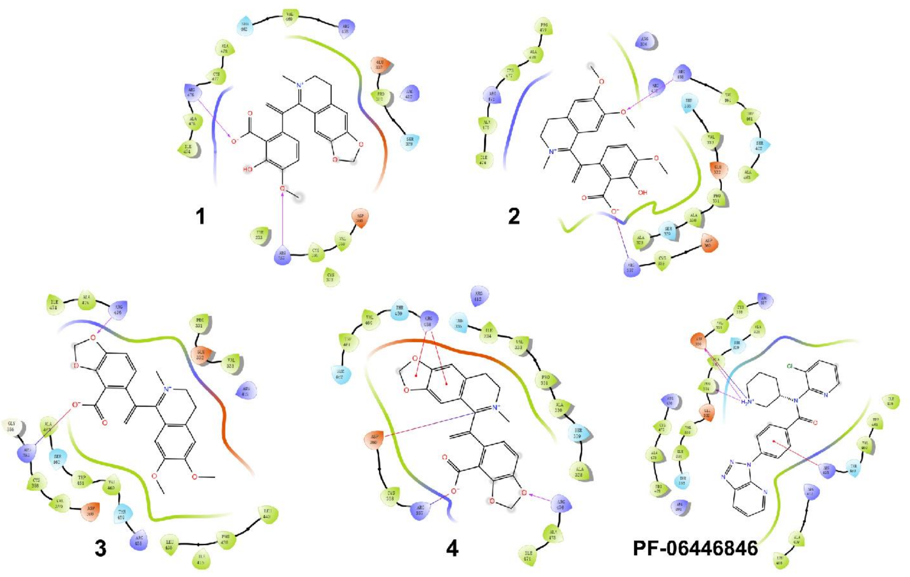

Figure S5. The 2D interacting mode of docking results of compounds **1-4** and PF-06446846 with PCSK9 (PDB ID: 6U3X).
